# From Cold to Hot: Nanozyme‐Based Strategies for Reprogramming the Tumour Immunoenvironment

**DOI:** 10.1111/cpr.70235

**Published:** 2026-05-24

**Authors:** Yue Wang, Miao Xu, Yongshun Wang, Xuan Lin, Qiang Zhang, Xiaoning Lin, Xin Wu, Cheng Zhang, Wenhua Huang, Jianlin Shen

**Affiliations:** ^1^ Central Laboratory, Affiliated Hospital of Putian University School of Basic Medicine, Putian University Putian Fujian China; ^2^ Key Laboratory of Translational Tumour Medicine in Fujian Province Putian University Putian Fujian China; ^3^ School of Basic Medicine Fujian Medical University Fuzhou Fujian China; ^4^ Department of Orthopedics Affiliated Hospital of Putian University Putian Fujian China; ^5^ Department of Orthopaedics, Zhongda Hospital School of Medicine, Southeast University Nanjing Jiangsu China; ^6^ Guangdong Engineering Research Center for Translation of Medical 3D Printing Application, National Key Discipline of Human Anatomy School of Basic Medical Sciences, Southern Medical University Guangzhou Guangdong China

**Keywords:** cold tumours, combination therapies, immunogenic cell death, nanozymes, tumour immunoenvironments

## Abstract

The immunosuppressive tumour immune microenvironment (TIME) is a fundamental barrier that renders “cold” tumours resistant to conventional cancer immunotherapies. Nanozymes, catalytic nanomaterials with enzyme‐mimicking activities, have emerged as powerful and versatile agents for reprogramming the TIME and igniting robust antitumour immunity. This review systematically elucidates how nanozymes, through their multi‐enzyme catalytic properties, orchestrate a multifaceted attack on the immunosuppressive TIME by regulating reactive oxygen species, alleviating hypoxia, depleting antioxidants, and inducing immunogenic cell death, ferroptosis, and cuproptosis. These catalytic actions collectively promote dendritic cell maturation, enhance cytotoxic T lymphocyte infiltration, and repolarise tumour‐associated macrophages toward an M1 phenotype, thereby effectively converting immunologically “cold” tumours into “hot” ones. Furthermore, we deeply analyse the synergistic potential of nanozymes when integrated with established therapies—including immune checkpoint blockade, phototherapy, sonodynamic therapy, chemotherapy, and radiotherapy. Finally, by discussing advanced bioengineered platforms and addressing the ongoing challenges related to biosafety and clinical translation, we envision that nanozyme‐based catalytic immunoengineering represents a paradigm‐shifting approach for next‐generation combinatorial cancer immunotherapy.

## Graphical abstract

Nanozymes, with multi‐enzyme catalytic activities, reprogram the immunosuppressive tumour microenvironment by inducing immunogenic cell death, relieving hypoxia, and depleting antioxidants, effectively converting immunologically “cold” tumours into “hot” ones to boost cancer immunotherapy efficacy.

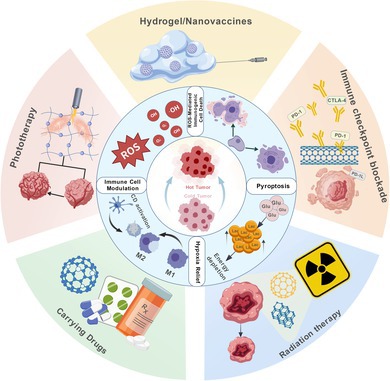

## 1 Introduction

Recent advances in nanotechnology have established nanomedicine as a frontier interdisciplinary field, which is now extensively applied across various stages of disease management—including prevention, diagnosis, monitoring, treatment, and tissue regeneration [1]. Current research in this domain focuses on areas such as hyperthermia, photodynamic therapy, nanozymes, and nano‐bio interactions [1]. Among these, nanozymes—artificial nanomaterials that mimic natural enzyme activities—have attracted significant interest. Owing to their high catalytic efficiency, stability, cost‐effectiveness, and low immunogenicity, they are increasingly being developed for innovative therapeutic and combination strategies against various diseases, such as cancer and inflammatory disorders [2, 3].

Since the concept of nanozymes was first introduced by Scrimin et al. in 2004, increasing attention has been paid to their therapeutic potential, especially in combination with other anticancer modalities [4]. Unlike conventional treatments, nanozymes can simulate multiple enzyme activities (e.g., peroxidase, superoxide dismutase, and glutathione oxidase) and dynamically respond to acidic, redox, and metabolic signals within the tumour microenvironment. These functionalities enable precise modulation of the immunosuppressive tumour milieu. Moreover, when combined with other modalities—notably phototherapy, chemotherapy, immune checkpoint blockade (ICB), and adoptive cell therapy—nanozymes have demonstrated considerable promise as immunomodulatory adjuvants [5, 6, 7].

Recent studies have further suggested that nanozymes can sensitise immunologically “cold” tumours to ICB by enhancing tumour immunogenicity and alleviating key suppressive barriers in the tumour microenvironment [8, 9, 10]. For example, a biomimetic Cu_2_O‐OMV nanozyme was shown to induce cuproptosis–pyroptosis crosstalk, enhance cytotoxic T‐lymphocyte infiltration, and markedly improve the therapeutic efficacy of αPD‐L1 treatment in renal carcinoma models [9]. Similarly, FeSA‐Ir@PF NSs triggered sequential ferroptosis and pyroptosis under light control, leading to robust multi‐immunogenic responses and substantially enhanced antitumour effects when combined with ICB [10]. Mechanistically, such synergy is mainly attributed to nanozyme‐driven reactive oxygen species (ROS) generation, immunogenic cell death (ICD), antigen release, and reversal of hypoxia‐ or metabolism‐related immunosuppression, which together promote dendritic‐cell activation and CD8^+^ T‐cell‐mediated antitumour immunity [8, 11, 12, 13].

The tumour immune microenvironment (TIME) plays a decisive role in cancer progression, therapeutic response, and patient prognosis. Although ICB therapy has revolutionised oncology, its efficacy remains limited in tumours with a “cold” phenotype, which are typically characterised by inadequate T‐cell infiltration, deficient IFN‐γ signalling, low PD‐L1 expression, and low tumour mutation burden [14]. More importantly, their poor responsiveness to immunotherapy also reflects deeper immunological defects, including insufficient priming and recruitment of tumour‐reactive T cells, impaired antigen presentation, and the establishment of a profoundly immunosuppressive microenvironment [15, 16]. In many cold tumours, low expression of T‐cell‐recruiting chemokines restricts the trafficking of effector lymphocytes [17, 18], while persistent antigen exposure, hypoxia, and metabolic stress drive infiltrating T cells toward an exhausted state marked by upregulation of inhibitory receptors such as PD‐1, LAG‐3, and TIM‐3, mitochondrial dysfunction, and reduced secretion of effector cytokines including IFN‐γ and TNF‐α [19, 20, 21]. In parallel, defects in IFN‐γ–JAK/STAT signalling and downregulation of MHC class I or other antigen‐processing machinery further weaken tumour antigen presentation and prevent the establishment of an inflamed microenvironment [22, 23]. Together, these features limit the effectiveness of PD‐1/PD‐L1 blockade. By contrast, “hot” tumours generally exhibit pre‐existing CD8^+^ T‐cell infiltration and a pro‐inflammatory immune contexture enriched in effector lymphocytes and M1‐like macrophages, thereby showing greater sensitivity to immunotherapy [15, 24, 25].

In this context, a central challenge in tumour immunology is to convert “immune‐desert” tumours into “immune‐active” ones to improve response rates to immunotherapy. Nanozymes have emerged as a promising strategy to overcome this limitation through multiple mechanisms, including ROS regulation, hypoxia alleviation, induction of ICD, and activation of metabolism‐associated cell death pathways such as ferroptosis and cuproptosis, thereby offering new opportunities for TIME reprogramming [26]. Among these mechanisms, the link between classical ICD and adaptive immune activation is best established, whereas the immunogenic roles of ferroptosis are more context‐dependent and those of cuproptosis remain emerging [27, 28].

Although numerous reviews have summarised the biomedical applications of nanozymes and explored their potential in cancer therapy and immunotherapy, a systematic integration from the perspective of TIME reprogramming and the conversion of “cold tumours” into “hot tumours” remains limited. Existing reviews have largely focused on the catalytic properties of nanozymes or on individual therapeutic mechanisms, often lacking a cohesive framework linking catalytic reactions, microenvironment remodelling, and immune activation. To clarify the positioning of the present review within the current research landscape, Table 1 compares representative recent reviews on nanozyme‐based cancer immunotherapy and TIME remodelling in terms of their organising principles, primary focus, and relative scope, while highlighting the distinctive contribution of this work.

**TABLE 1:** Representative recent reviews relevant to nanozyme‐based cancer immunotherapy and TIME remodelling.

Recent review	Organising principle	Primary focus	Relative scope or aspects less emphasised	How the present Review differs
Fu et al., ACS Nano, 2024 [29]	Material‐ and enzyme‐activity‐centered	Transition‐metal‐based nanozymes; catalytic mechanisms (CAT, POD, OXD, SOD); activity regulation and broad anticancer applications	Emphasises materials and catalytic activities, with limited focus on TIME remodelling and immune phenotype conversion	Connects catalytic reactions with TIME reprogramming, ICD, and cold‐to‐hot tumour conversion
Xu et al., J. Mater. Chem. B, 2024 [30]	Metabolism–immunotherapy interaction‐centered	Nanozyme‐mediated metabolic disruption and its synergy with cancer immunotherapy	Focuses on metabolic regulation and therapeutic synergy, with less emphasis on broader immune remodelling	Extends metabolism‐centered discussion to ICD, ferroptosis, immune modulation, and combination therapy
He and Zhang, Nanoscale, 2025 [31]	Immune microenvironment‐centered, multi‐disease scope	Nanozyme‐mediated immunoregulation across cancer, inflammation, infection	Broad overview of immune microenvironment remodelling, but less centered on tumour‐specific cold‐to‐hot conversion	Provides a tumour‐focused framework linking catalytic activity to TIME reprogramming
Iroegbu et al., Nanomedicine, 2025 [32]	Design‐ and engineering‐centered	Structural design, stimulus responsiveness, delivery strategies, and biosafety of engineered nanozymes	Focuses on nanozyme design and engineering, with less explicit mapping to immune outcomes	Bridges nanozyme function with TIME modulation and antitumour immune activation
Zhang et al., Coord. Chem. Rev., 2025 [33]	Structure–function–therapy integration	Advanced nanozyme design and targeted oncotherapy, including synergistic immunotherapy	Covers advanced nanozyme systems and synergistic therapy, but less emphasises immune phenotype transformation	Integrates catalytic reactions with immune–metabolic remodelling and cold‐to‐hot conversion
Present review	Barrier‐oriented framework for TIME reprogramming and cold‐to‐hot tumour conversion	TIME remodelling via ROS‐mediated ICD, ferroptosis/metabolic reprogramming, hypoxia/redox normalisation, immune‐cell modulation, and rational combination therapy	N/A	Key features: barrier‐oriented cold‐to‐hot conversion, integration of catalytic–immune–metabolic mechanisms, coordinated discussion of cell death and immune activation, and emphasis on rational combinational immunotherapy

Abbreviations: CAT, catalase; ICD, immunogenic cell death; OXD, oxidase; POD, peroxidase; ROS, reactive oxygen species; SOD, superoxide dismutase; TIME, tumour immune microenvironment.

**FIGURE 1:**
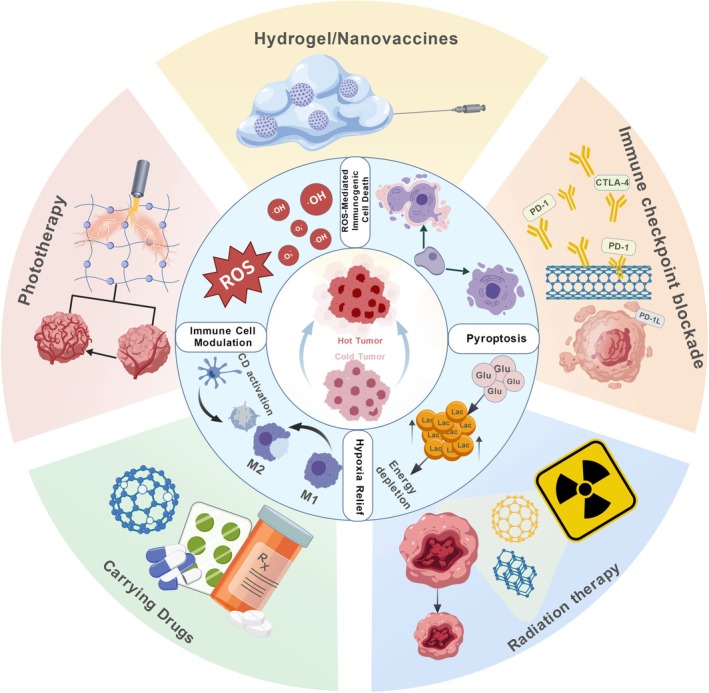
Schematic overview of nanozyme applications in tumour immunotherapy. The illustration highlights the role of nanozymes in modulating the tumour microenvironment—affecting immune cells, reactive oxygen species (ROS), hypoxia, and pyroptosis—and their combinatorial use with other therapeutic strategies, such as immune checkpoint blockade, phototherapy, radiation therapy, drug delivery, and other bioengineered platforms. Created with BioGDP.com.

Against this backdrop, the present review provides an integrative perspective on nanozyme‐based tumour immunotherapy. We systematically summarise recent advances in nanozyme‐mediated regulation of the TIME, with particular emphasis on their roles in promoting the conversion of cold tumours into hot tumours. We further propose a catalytic–immune interaction framework to illustrate how nanozyme‐mediated processes, including ROS modulation, hypoxia alleviation, and antioxidant system depletion, synergistically remodel the TIME and promote ICD and immune‐cell activation. In addition, we discuss the synergistic potential of nanozymes in combination with ICB, photodynamic/photothermal therapy (PTT), chemotherapy, and radiotherapy (Figure 1). By integrating catalytic mechanisms, microenvironment modulation, and immunotherapeutic strategies, and by discussing current translational challenges and future directions, this review aims to provide a systematic reference for the development of nanozyme‐based cancer immunotherapy.

## 2 Properties and Mechanisms of Nanozymes

### 2.1 Definition and Properties of Nanozymes

Nanozymes are a class of artificial enzymes with unique physicochemical properties and catalytic functions at the nanoscale, which have garnered widespread attention in biomedical and related fields. These nanomaterials possess significant advantages over natural enzymes and traditional small‐molecule drugs, including high catalytic efficiency, superior structural stability, low production cost, and excellent scalability [34, 35]. Typically composed of inorganic nanomaterials, nanozymes exhibit enzyme‐like activity at the nanoscale, mimicking natural enzymatic catalysis while offering exceptional stability and economic benefits. Their defining characteristics encompass high catalytic activity, structural tunability, cascade reaction capability, low immunogenicity, and good biocompatibility.

However, it should be emphasised that the catalytic efficiency and biocompatibility of nanozymes are not intrinsic or universal properties, but are highly dependent on their physicochemical parameters, including size, composition, valence state, crystal structure, and surface functionalisation [36]. For instance, catalytic activity varies markedly across material systems: transition metal oxides often rely on Fenton‐like reactions under acidic pH, noble metals (e.g., Au, Pt) mediate surface electron transfer with limited substrate specificity, and carbon‐based nanozymes leverage defect structures and heteroatom doping, though their turnover efficiency is generally lower than that of natural enzymes [37, 38, 39, 40].

From a biosafety perspective, nanozymes also display pronounced context dependence. Metal ions released from nanozymes can induce oxidative stress and activate inflammatory pathways (e.g., NF‐κB), while excessive ROS may damage biomolecules [41, 42, 43]. Furthermore, the adsorption of plasma proteins onto nanozyme surfaces—forming a protein corona—can alter their biological identity and enhance clearance by the mononuclear phagocyte system (MPS), thereby increasing immunogenicity [44, 45]. Importantly, surface engineering strategies such as PEGylation or biomimetic cell membrane coating can effectively reduce these adverse effects and improve biocompatibility [46]. Therefore, the evaluation of nanozyme performance must be considered in the context of specific material systems and biological microenvironments, rather than being generalised as inherently possessing uniformly high catalytic efficiency or low immunogenicity.

To address this context‐dependent variability, current research emphasises the precise engineering of catalytic sites, known as active site engineering. This approach involves customising specific nanostructures, such as single‐atom nanozymes (SAzymes), high‐entropy nanozymes (HEzymes), and defect‐engineered materials. These advanced designs aim to replicate the sophisticated active centers of natural enzymes, thereby enhancing catalytic efficiency and substrate specificity. For instance, SAzymes utilise isolated metal atoms as catalytic centers. This atomic dispersion enables fine control over the coordination environment and direct regulation of the electronic structure, which significantly improves catalytic performance [47].

Compared to conventional enzymes and small‐molecule drugs, nanozymes offer additional therapeutic benefits, such as enhanced accumulation at disease sites, sustained catalytic action, and reduced treatment‐related side effects [35, 48, 49]. Moreover, their catalytic function can be easily modulated by adjusting the nanostructure or the catalytic microenvironment, making them highly promising for applications in disease diagnosis and therapy [2]. Quantitative kinetic analysis further substantiates the functional advantages of nanozymes. Specifically, while natural enzymes are characterised by high catalytic efficiency (Vmax) and strong substrate affinity (exemplified by a low Michaelis constant, Km), nanozymes typically excel in operational stability and cost‐effectiveness. Catalytic metrics, such as Vmax and Km, are crucial for evaluating catalytic capability; therefore, comparative kinetic analysis provides scientific justification for the superiority of nanozymes in harsh environments like the tumour microenvironment [50].

Importantly, this suitability for harsh tumour conditions is not only a matter of kinetic performance; it also reflects the ability of nanozymes to respond to characteristic biochemical cues within the tumour microenvironment. Nanozymes can be engineered to exploit characteristic biochemical cues within the tumour microenvironment, including acidic pH, elevated H_2_O_2_ levels, and redox imbalance, thereby achieving preferential activation at tumour sites and reducing off‐target effects. Unlike many natural enzymes, which are prone to denaturation or loss of activity under harsh tumour conditions, certain nanozymes exhibit pH‐dependent catalytic behaviour and remain highly active mainly in the acidic tumour microenvironment while being relatively less active in normal tissues [51]. In addition, iron‐ or copper‐based nanozymes can utilise endogenous H_2_O_2_ to drive Fenton or Fenton‐like reactions and generate cytotoxic hydroxyl radicals, whereas glutathione (GSH)‐responsive systems can consume GSH, inhibit its regeneration, or undergo tumour‐triggered biodegradation to enhance redox disruption [52, 53, 54]. Through these tumour microenvironment‐responsive processes, nanozymes not merely mediate localised tumour killing but also amplify oxidative stress, weaken antioxidant defences, and help remodel immunosuppressive conditions, thereby providing an important physicochemical basis for subsequent TIME reprogramming.

### 2.2 Classification and Mechanisms of Action of Nanozymes

**FIGURE 2:**
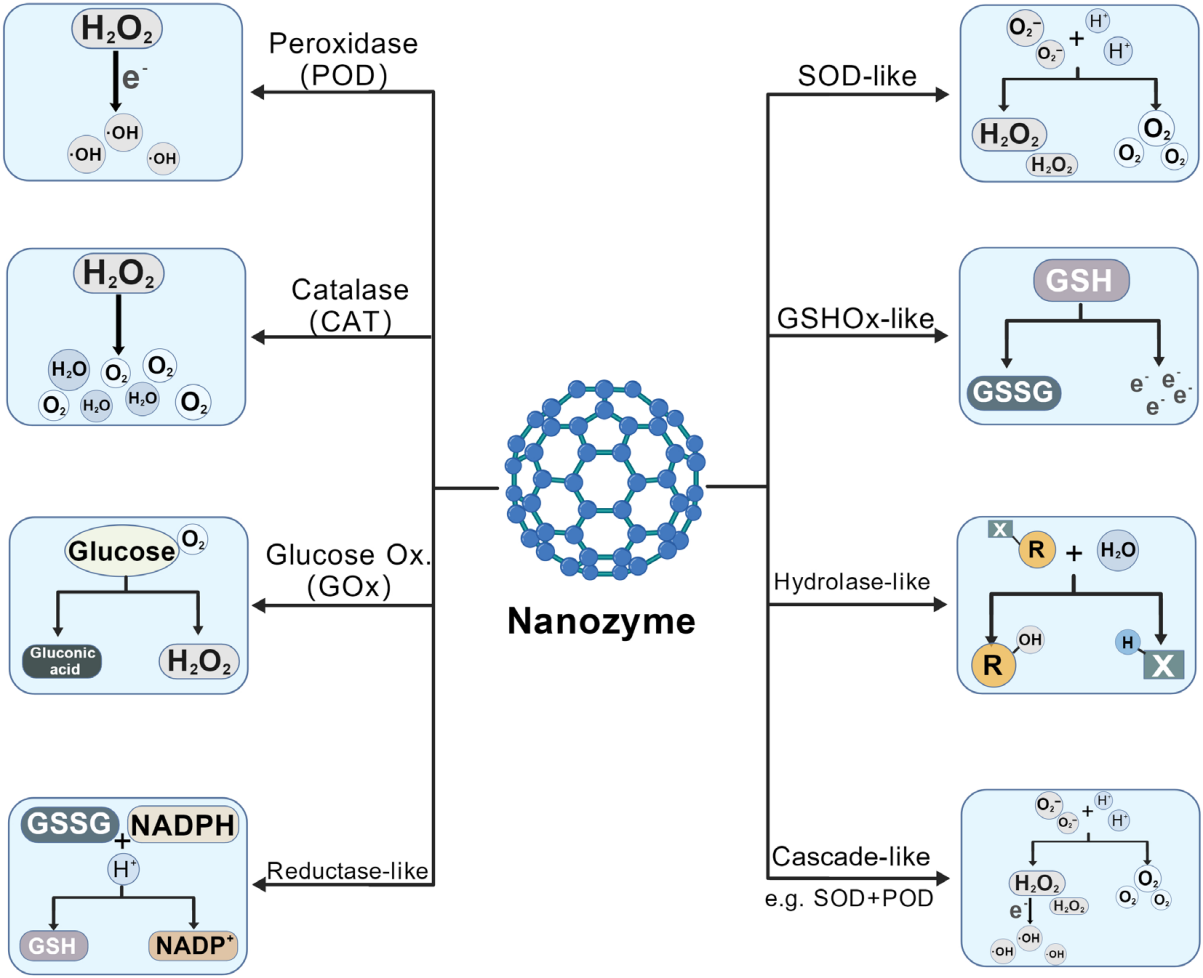
Schematic diagram of nanozyme‐mimicking enzyme activities. This figure summarises representative catalytic functions of nanozymes, including POD‐like (catalysing oxidation), CAT‐like (decomposing H_2_O_2_), OXD‐like (electron transfer), SOD‐like, hydrolase‐like, reductase‐like, and cascade‐like activities. Created with BioGDP.com.

Various types of nanozymes have been developed by mimicking the functions of natural enzymes, including those exhibiting peroxidase (POD)‐like, catalase (CAT)‐like, glucose oxidase (GOx)‐like, and superoxide dismutase (SOD)‐like catalytic activities [29, 55, 56, 57]. Functionally, nanozymes can be categorised as either antioxidant or pro‐oxidant enzymes. Antioxidant nanozymes primarily scavenge excessive ROS to mitigate oxidative stress‐related damage, while pro‐oxidant nanozymes generate ROS to selectively induce tumour cell death—a mechanism widely utilised in cancer therapy [58]. Figure 2 summarises representative types of nanozyme catalysts based on this functional classification. Additionally, nanozymes can be categorised according to their material composition, such as metal oxide‐based, noble metal‐based, and carbon‐based nanozymes.

The diverse enzyme‐mimicking activities of nanozymes, along with their corresponding catalytic mechanisms and applications in tumour therapy, are systematically summarised in Table 2. These activities range from generating cytotoxic radicals (e.g., POD‐like) and modulating tumour hypoxia (e.g., CAT‐like) to disrupting metabolic pathways (e.g., GOx‐like) and amplifying oxidative stress (e.g., GSHOx‐like). This functional versatility enables nanozymes to intervene in multiple biochemical processes within the tumour microenvironment.

**TABLE 2:** Representative nanozyme activities, catalytic actions, and immunomodulatory effects in TIME reprogramming.

Nanozyme type	Mimicked enzyme activity	Catalytic mechanism	Example applications	Immunomodulatory effects
Peroxidase‐like (POD)	H_2_O_2_ → •OH (Fenton/Fenton‐like reaction)	Promote electron transfer; catalyse substrate oxidative decomposition	Tumour ROS therapy	Induces ROS‐mediated ICD and DAMP release, thereby promoting dendritic‐cell maturation and T‐cell priming
Catalase‐like (CAT)	2H_2_O_2_ → 2H_2_O + O_2_	Decomposition of H_2_O_2_; alleviates hypoxia	TME regulation, antioxidant protection	Relieves tumour hypoxia and HIF‐1α‐associated immunosuppression, thereby enhancing immune‐cell infiltration and antitumour immune activation
Glucose Oxidase‐like (GOx)	Glucose + O_2_ → Gluconic acid + H_2_O_2_	Depletes glucose; generates endogenous H_2_O_2_	Metabolic intervention therapy	Depletes glucose and generates endogenous H_2_O_2_, thereby amplifying oxidative stress, promoting ICD, and enhancing sensitivity to immunotherapy
Superoxide Dismutase (SOD)	2O_2_^−^ + 2H^+^ → H_2_O_2_ + O_2_	Scavenges superoxide anions; modulates redox balance	Oxidative stress intervention	Modulates ROS homeostasis and, in multienzyme systems, supports downstream catalytic cascades that contribute to TIME normalisation and immune activation
Glutathione Oxidase (GSHOx)	2GSH → GSSG +2e^−^	Oxidises GSH, disrupts antioxidant defence	Enhancement of POD efficacy	Depletes intracellular GSH, disrupts redox buffering, amplifies ROS accumulation, and sensitises tumours to ICD and ferroptosis‐related immune activation
Hydrolase‐like	R‐X + H_2_O → R‐OH + HR	Mimics lipases, alkaline phosphatases, proteases, etc.	Drug release, biodegradation, biosignal amplification	Facilitates stimulus‐responsive drug release or substrate degradation, thereby improving immune‐cell infiltration and therapeutic responsiveness
Reductase‐like	NO_3_^−^ + 2e^−^ + 2H^+^ → NO_2_^−^ + H_2_O	Reduction of substrates via electron transfer	Antioxidant therapy, signal transduction, drug delivery	Regulates redox‐active substrates and signalling molecules, thereby modulating inflammatory signalling and immune responsiveness
Cascade‐like	Serial or coexistent multiple enzyme activities	Materials possessing two or more types of enzyme‐like activity simultaneously	Self‐supplied substrate catalysis (e.g., GOx produces for POD use)	Integrates multiple enzyme‐mimicking activities to simultaneously induce ICD, relieve immunosuppression, and promote the conversion of “cold” tumours into “hot” tumours

Abbreviations: DAMP, damage‐associated molecular patterns; GOx, glucose oxidase; GSH, glutathione; HIF‐1α, hypoxia‐inducible factor‐1 alpha; ICD, immunogenic cell death; POD, peroxidase; ROS, reactive oxygen species; TIME, tumour immune microenvironment; TME, tumour microenvironment.

Importantly, these enzyme‐mimicking activities do not contribute equally to TIME regulation, but instead act through partially distinct yet cooperative mechanisms. Broadly, POD‐like, glutathione oxidase (GSHOx)‐like, and GOx‐like nanozymes mainly participate in a pro‐oxidant immunogenic activation process, whereas CAT‐like and SOD‐like nanozymes are more closely associated with the relief of immunosuppressive barriers in the tumour microenvironment [59]. POD‐like nanozymes act as major pro‐oxidant effectors by catalysing Fenton or Fenton‐like reactions that convert endogenous H_2_O_2_ into highly reactive hydroxyl radicals (·OH), thereby promoting oxidative damage, ICD, and the release of damage‐associated molecular patterns (DAMPs) [60, 61]. GSHOx‐like activity complements this process by depleting intracellular GSH, disrupting redox homeostasis, and sensitising tumour cells to ROS‐dependent ICD and ferroptosis [62]. In parallel, GOx‐like nanozymes provide metabolic and substrate support for this pro‐oxidant cascade by consuming glucose while generating gluconic acid and endogenous H_2_O_2_, thereby coupling starvation effects with sustained substrate supply for downstream POD‐like catalysis [63].

By contrast, CAT‐like nanozymes mainly regulate the hypoxic and immunosuppressive axis of the TIME. Because hypoxia‐driven HIF‐1α signalling is closely associated with M2‐like macrophage polarisation, impaired effector T‐cell function, and immune evasion [64], CAT‐like nanozymes can alleviate tumour hypoxia by decomposing H_2_O_2_ into H_2_O and O_2_ and may thereby attenuate HIF‐1α‐related immunosuppressive signalling, help reverse hypoxia‐driven macrophage polarisation, and improve the activity of tumour‐infiltrating immune cells [65]. SOD‐like nanozymes may further support this process by scavenging excess superoxide anions (O_2_^−^) to reduce nonspecific oxidative stress while, in some systems, also generating H_2_O_2_ and O_2_ for subsequent cascade reactions and improved tumour microenvironment oxygenation [66]. Therefore, from a TIME‐regulation perspective, nanozyme classification should be viewed not merely as a catalogue of catalytic types, but also as a functional framework linking distinct enzyme‐like activities to specific immunoregulatory outcomes, including ROS‐driven ICD, antioxidant depletion, metabolic intervention, hypoxia relief, and immune‐cell repolarisation.

The catalytic mechanism of nanozymes is closely associated with their nanostructure. Specifically, different elemental compositions determine distinct electronic structures and reaction pathways, while the nanostructure itself facilitates electron transfer and modulates catalytic efficiency [67]. Moreover, factors such as material composition, surface modification, and structural defects can significantly influence nanozyme performance. For instance, metal oxide nanozymes (such as iron oxide nanoparticles) exhibit POD‐like activity, catalysing the Fenton reaction to generate hydroxyl radicals (•OH). These radicals induce tumour cell membrane damage and macromolecular degradation—including proteins and nucleic acids—ultimately leading to cell death [68, 69]. In contrast, noble metal nanozymes (e.g., gold nanoparticles) typically act as pro‐oxidants, catalysing ROS generation while maintaining their structural integrity under catalytic conditions [70]. Although both categories can promote tumour cell death via ROS generation, metal oxide nanozymes generally exhibit a wider spectrum of catalytic types and hold broader potential for practical applications.

## 3 Nanozyme‐Based Strategies for Reprogramming the Tumour Immune Microenvironment (TIME)

### 3.1 Challenges of the TIME

The tumour microenvironment comprises both non‐cancerous cellular and acellular components within and surrounding a tumour, including immune cells, stromal cells, the extracellular matrix, and soluble mediators. This dynamic milieu critically influences tumour initiation, progression, metastasis, and therapeutic responses [71]. Accordingly, targeting the tumour microenvironment has emerged as a pivotal strategy in oncology research. Accumulating evidence further indicates that tumour microenvironment modulators play essential roles in immune surveillance, immune escape, and the clinical efficacy of anticancer treatments [72, 73, 74].

The cellular composition of the tumour microenvironment is highly heterogeneous, varying with tumour type, stage, genetic alterations, and patient‐specific factors [24]. One major immunosuppressive feature, hypoxia, impairs mitochondrial function in T cells, promoting T cell exhaustion and diminished responses to immunotherapy. Consequently, strategies that enhance oxygen availability or modulate cellular metabolism represent promising avenues to restore antitumour immunity and improve therapeutic outcomes [75].

Tumours are commonly categorised based on immune contexture as “hot” (immune‐inflamed) or “cold” (immune‐desert) phenotypes [14]. Hot tumours are characterised by abundant CD8^+^ T cell infiltration and elevated expression of immune checkpoint molecules such as PD‐L1. By contrast, cold tumours lack immune stimulatory signals, tumour neoantigens, and efficient antigen‐presenting cell recruitment, collectively resulting in inadequate T cell activation and poor response to immunotherapies [9, 76]. Therefore, overcoming the multifactorial immunosuppression within the TIME is critically important.

**FIGURE 3:**
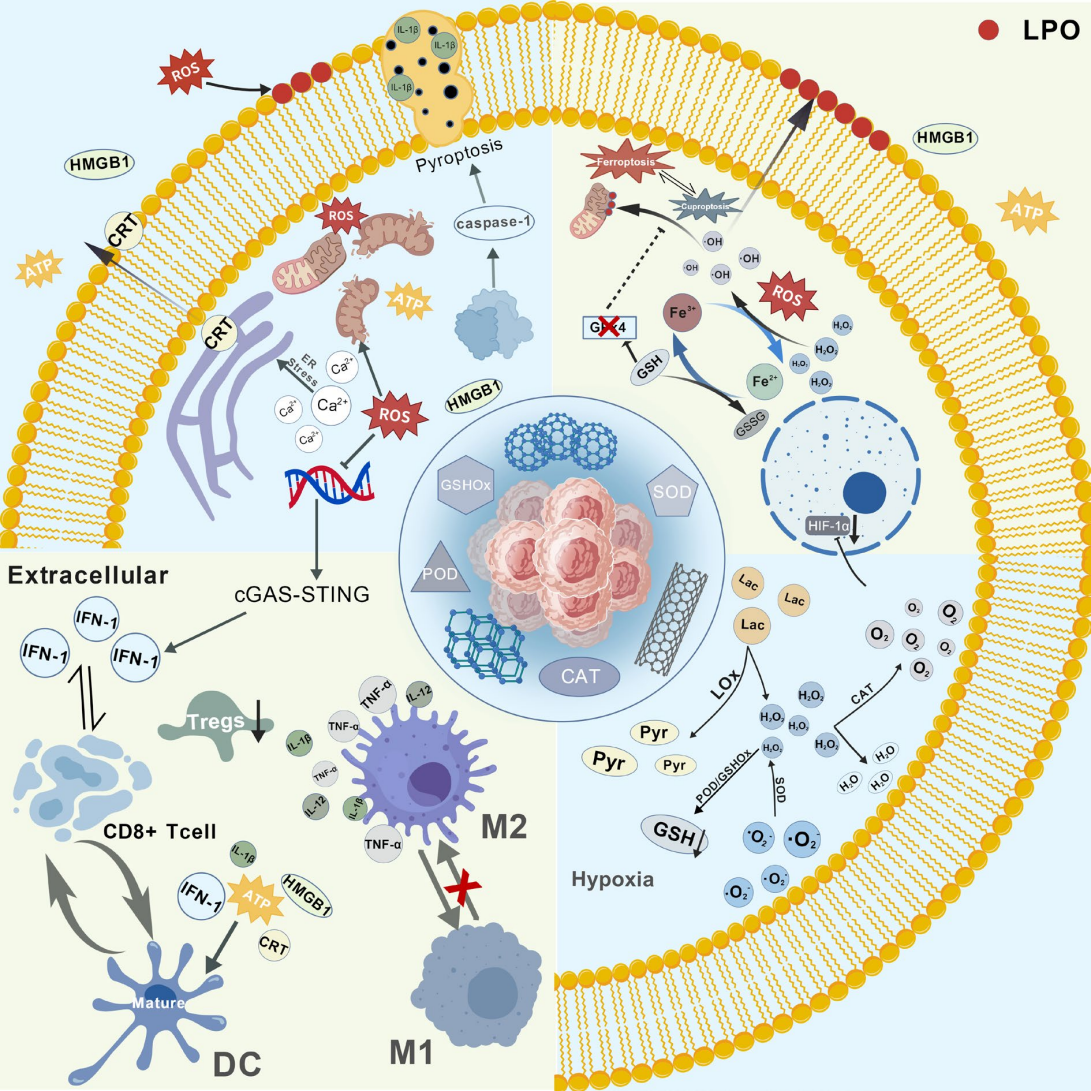
Mechanisms of nanozyme‐mediated remodelling of the tumour immune microenvironment (TIME). Nanozymes with multi‐enzyme activities (e.g., POD, CAT, SOD, GSHOx, LOX) drive several key processes: ROS‐mediated immunogenic cell death, ferroptosis and metabolic reprogramming, hypoxia relief and redox modulation, as well as immune cell modulation. These actions promote immunogenic cell death and damage‐associated molecular patterns (DAMPs) release, activate the cGAS–STING pathway, enhance dendritic cell (DC) maturation and CD8^+^ T cell responses, and repolarise tumour‐associated macrophages (TAMs) toward the M1 phenotype. These mechanisms convert the TIME to boost immunotherapy. Created with BioGDP.com.

In this context, nanozyme‐based approaches hold considerable promise for reprogramming the TIME. These multifunctional systems operate through multiple mechanisms: alleviating hypoxia, modulating redox homeostasis, inducing ICD, and remodelling immune cell populations (Figure 3). By mimicking natural enzymatic activities and enabling targeted metabolic interventions, nanozymes facilitate the conversion of immunologically “cold” tumours into “hot” phenotypes, thereby offering a viable strategy to augment the efficacy of cancer immunotherapies.

### 3.2 ROS‐Mediated Immunogenic Cell Death (ICD)

ROS are pivotal in inducing ICD, a process that activates adaptive immune responses against tumours. Mechanistically, ROS‐induced oxidative stress triggers endoplasmic reticulum (ER) stress, mitochondrial damage, and lipid peroxidation (LPO), ultimately leading to the exposure of DAMPs—including calreticulin—and the release of ATP and high‐mobility group box 1 (HMGB1) [77]. Beyond these DAMPs, ROS can activate the NLRP3 inflammasome, promoting caspase‐1‐dependent pyroptosis and the release of interleukin‐1β; this process subsequently enhances dendritic cell (DC) maturation and T cell priming [78]. Concurrently, ROS‐mediated DNA damage activates the STING pathway via cytosolic DNA sensing, thereby amplifying type I interferon responses and CD8^+^ T cell cross‐priming [79]. As a representative example, MnO_2_@CeOx‐GAMP nanozymes have been shown to boost radiotherapy‐induced STING activation through dual enzyme‐mimetic activities and enhanced cyclic GMP‐AMP production, collectively amplifying antitumour immunity [80]. Despite these mechanisms, the efficacy of ROS‐dependent therapies is often limited by insufficient endogenous H_2_O_2_ levels and tumour hypoxia.

To overcome these limitations, nanozymes have emerged as a promising strategy for the spatiotemporal control of ROS levels within the tumour microenvironment. By leveraging their enzyme‐mimetic activities and tumour microenvironment‐responsive properties, nanozymes can enhance ROS generation, alleviate hypoxia, and disrupt redox homeostasis, thereby collectively facilitating robust antitumour immunity.

A notable example is a tumour microenvironment‐responsive CaCO_3_@Pt‐TiO_2_ (CaPT) nanocomposite enhances sonodynamic therapy (SDT) under ultrasound irradiation. In this system, platinum (Pt) nanoparticles augment the sonocatalytic activity of TiO_2_, leading to substantial ROS generation while also decomposing H_2_O_2_ to alleviate hypoxia. Simultaneously, the acid‐triggered release of Ca^2+^ induces calcium overload, which synergises with ROS to amplify oxidative stress and trigger robust ICD. This cascade effectively converts immunologically “cold” tumours into “hot” ones, thereby improving responses to immunotherapy [81].

Another representative nanozyme, IMZF, disassembles in the tumour microenvironment to sequentially mimic SOD, CAT, POD, and GSHOx activities. Through these actions, it alleviates hypoxia, polarises M2 macrophages to the M1 phenotype, depletes GSH, and generates lethal levels of ROS, thereby inducing both ICD and pyroptosis. Consequently, this promotes DC maturation, enhances lymphocyte infiltration, and suppresses both primary and metastatic tumours [82]. Similarly, copper‐based SAzymes provoke apoptosis through GSHOx‐ and POD‐mimetic cascade reactions that disrupt redox homeostasis [83, 84]. Beyond single‐element systems, heterostructures such as UCNPs@Cu‐PN‐g‐C_3_N4 integrate ROS generation with photodynamic and photothermal effects to enhance tumour immunogenicity [85].

The nanozyme toolkit further includes a photo−/metallo‐immunotherapeutic agent featuring an AuPt heterostructure and Pt nanoclusters, which generates robust ROS under near‐infrared (NIR) light and releases Pt^2+^ ions to synergistically induce ICD [86]. Additionally, bismuth‐based ternary heterojunctions (BBOP) enhance sonocatalytic ROS production to trigger ICD and PANoptosis [87], whereas copper‐doped BiSe_x_ (CBS) nanozymes induce apoptosis and cuproptosis, an effect augmented by NIR irradiation [88]. Moreover, manganese platinum (MnPt) nanozymes with dual ROS‐generating capabilities facilitate chemodynamic therapy (CDT) and ICD [89].

Advancing in complexity, nanozymes such as PtMnIr operate through catalytic feedback loops, continuously producing ROS and depleting GSH to promote ferroptosis and apoptosis [90]. Similarly, HEzymes exhibit enhanced POD‐like activity and photothermal effects that reinforce ROS generation and M2‐to‐M1 macrophage repolarisation [91]. In an alternative strategy, vanadium nitride quantum dots act as nitric oxide donors, generating reactive nitrogen species to enhance ICD [92]. Furthermore, manganese‐based SAzymes and genetically engineered cascade nanozymes (e.g., gCM@MnAu) activate the cGAS–STING and type I interferon pathways, thereby amplifying immune responses [61]. Finally, an ultrasound‐driven piezoelectric nanozyme (BFTM) enhances ROS generation, triggering pyroptosis via the caspase‐1/GSDMD pathway and restoring T‐cell receptor signalling in CD8^+^ T cells [93].

In summary, these nanozyme‐based strategies collectively exemplify a multifaceted approach to reprogramming the immunosuppressive TIME. Through enhanced and sustained ROS production, modulation of hypoxia, depletion of antioxidants, and engagement of multiple ICD pathways, nanozymes effectively promote the transition from immunologically “cold” to “hot” tumours. This integrated action thus overcomes critical barriers to effective anticancer immunotherapy.

### 3.3 Ferroptosis and Metabolic Reprogramming

Ferroptosis is an iron‐dependent, non‐apoptotic form of cell death characterised by the accumulation of ROS and LPO. Given its capacity to trigger ICD and modulate the immunosuppressive TIME, this process has emerged as a significant therapeutic mechanism [94]. Notably, nanozymes offer a versatile catalytic platform for inducing ferroptosis by disrupting redox homeostasis, depleting antioxidant reserves, and generating hydroxyl radicals via Fenton or Fenton‐like reactions [95].

Building on this foundation, several iron‐based nanozymes—including HEzymes and MnFe‐based metal–organic frameworks (MnFe‐MOFs)—exhibit enhanced POD‐like activity, which promotes ferroptosis through sustained ROS production [91, 96]. These nanozymes possess diverse enzymatic activities, such as POD‐like, oxidase‐like, and GSHOx‐like functions, enabling concurrent ROS generation and disruption of tumour microenvironment homeostasis [97]. Furthermore, by leveraging Fenton chemistry, they augment tumour ferroptosis through multiple actions: modulating iron species, executing LPO, oxidising GSH, and inducing lysosomal destabilisation [98, 99, 100, 101].

The underlying mechanism involves the cyclic reduction of Fe^3+^ to Fe^2+^ ions by several iron‐based nanozymes, a process concomitant with ROS generation [102]. Following endocytic uptake and the consequent rise in intracellular iron levels, these nanozymes generate ·OH radicals. These reactive species subsequently attack phospholipids, membrane receptors, and polyunsaturated fatty acid side chains within macromolecules. The resulting ROS accumulation oxidises the biomembrane, initiating LPO [103], which in turn alters membrane fluidity and permeability, ultimately leading to tumour cell death. Concurrently, GSH is depleted during the reduction of Fe^3+^ to Fe^2+^, yielding oxidised GSH (GSSG); this depletion inhibits the GPx4 pathway, thereby further enhancing ferroptosis [104, 105]. For instance, a FeCo/Fe‐Co bimetallic atom nanozyme (FeCo/Fe‐Co DAzyme/PL) co‐loaded with lipoxygenase and phospholipase A2 not only simulates initial immunogenic tumour ferroptosis but also generates substantial ROS, depletes GSH, and induces LPO. This combined activity upregulates arachidonic acid expression and, together with IFN‐γ, cooperatively induces ACSL4‐mediated tumour ferroptosis [103].

Beyond ferroptosis induction alone, its therapeutic potential can be amplified by co‐activating complementary cell death pathways. For example, certain nanozymes can concurrently trigger cuproptosis, a copper‐dependent process linked to disrupted mitochondrial lipoylation [106]. Copper‐based nanozymes (CBS) exploit this pathway to reshape tumour metabolism and potentiate anti‐tumour immunity [107, 108]. Separately, vanadium nitride quantum dots induce ferroptosis while simultaneously releasing nitric oxide, thereby activating reactive nitrogen species pathways and enhancing ICD [92].

Besides direct tumour killing, ferroptosis exerts profound crosstalk with immune cells within the TIME, thereby influencing the efficacy of antitumour immunity. First, ferroptotic cells release DAMPs, such as HMGB1, as well as other oxidation‐associated signals, which can be sensed by immune cells and contribute to macrophage polarisation within the TIME [109, 110]. M1‐type tumour‐associated macrophages (TAMs), in turn, can support CD8^+^ cytotoxic T lymphocyte (CTL) activation [111]. Reciprocal crosstalk between ferroptotic tumour cells and macrophages may further reinforce a pro‐inflammatory microenvironment and promote ferroptosis‐associated antitumour immunity [110, 112]. By contrast, tumour‐intrinsic signalling such as Kirsten rat sarcoma 12D may contribute to polarisation toward the M2 phenotype81, and M2‐type TAMs can indirectly restrain ferroptosis by suppressing CTL activity or upregulating immunosuppressive molecules such as PD‐L1, thereby protecting tumour cells and maintaining an immunosuppressive microenvironment [113]. Ferroptosis also appears to influence the homeostasis and function of regulatory T cells (Tregs). Available evidence, although partly derived from non‐tumour settings, suggests that Tregs are sensitive to ferroptotic stress, whereas ferroptosis‐suppressive mechanisms, including m6A‐dependent regulation of genes such as SLC7A11, may help preserve their immunosuppressive function [114, 115]. Collectively, these findings indicate that ferroptosis is not merely a tumour‐cell‐intrinsic death program, but also an immunoregulatory process that shapes the balance between pro‐inflammatory TAM/CTL responses and Treg‐mediated suppression within the TIME. These immune consequences also help explain why ferroptosis‐inducing nanozyme platforms can synergise with immunotherapy.

The strategic integration of ferroptosis inducers with immunotherapy represents a promising approach to reverse TIME immunosuppression. Specifically, ferroptosis‐driven ICD promotes DC maturation, enhances T‐cell infiltration, and stimulates cytokine release, collectively facilitating the conversion of immunologically “cold” tumours to “hot” ones [116, 117]. Moreover, these inducers, alongside other metabolic interventions, not only direct tumour killing but also drive immune remodelling by increasing antigen release, promoting DC maturation, and downregulating PD‐L1 expression, thereby creating synergies with immunotherapies [99, 107, 117]. Consequently, combining ferroptosis‐based therapies with ICB has demonstrated potent antitumour efficacy in both metastatic and abscopal models [118, 119], underscoring the broad translational potential of nanozyme‐mediated metabolic reprogramming in cancer immunotherapy.

Importantly, the links between the cell‐death‐associated mechanisms discussed here and adaptive antitumour immunity are not equally established. Classical ICD remains the clearest and best‐supported framework for inducing dendritic‐cell activation and subsequent CD8^+^ T‐cell priming [60]. Ferroptosis has also been increasingly associated with antitumour immune activation, although its immunogenicity appears to be context‐dependent rather than universal [28]. By contrast, cuproptosis remains a newer and less well‐defined mechanism. Although emerging preclinical studies suggest that it may contribute to TIME remodelling, its direct and generalizable role in efficient antigen presentation and durable T‐cell priming is still being clarified [27, 120]. Moreover, many current nanozyme systems appear to co‐induce cuproptosis together with ferroptosis or ROS‐driven ICD, which makes pathway‐specific attribution more difficult [121, 122]. Therefore, ferroptosis may currently be viewed as an increasingly supported but context‐dependent immunogenic pathway, whereas the adaptive immune significance of cuproptosis should still be presented more cautiously.

### 3.4 Hypoxia Relief and Redox Modulation

Hypoxia, a hallmark of the tumour microenvironment, not only compromises the efficacy of oxygen‐dependent therapies but also fosters an immunosuppressive milieu by impairing effector T cell function and promoting immune escape through HIF‐driven pathways [123]. Nanozymes with CAT‐like activity can decompose H_2_O_2_ into O_2_ and have therefore been explored as oxygen‐generating components for hypoxia modulation in tumour therapy [124, 125, 126]. However, this effect should not be overstated. Although H_2_O_2_ levels are generally elevated in tumours relative to normal tissues, endogenous H_2_O_2_ may still be insufficient to sustain robust CAT‐like catalysis, particularly in deeply hypoxic tumour regions [56, 127, 128]. In addition, the oxygen‐generating performance of CAT‐like nanozymes is highly dependent on material composition, active‐site structure, catalytic kinetics, local pH, and substrate availability [56, 127]. Therefore, CAT‐like nanozymes may partially improve local oxygenation, but are often insufficient by themselves to fully reverse severe tumour hypoxia.

To overcome this limitation, recent studies have increasingly incorporated CAT‐like nanozymes into cascade‐amplified or multimodal systems. In particular, coupling CAT‐like nanozymes with GOx or GOx‐like activity can supplement intratumoural H_2_O_2_ production and support sustained catalytic cycling while also enabling starvation‐based metabolic intervention. For example, Janus‐type tBT@PANI‐MnO_2_ heterostructures exploit GOx/CAT cascade catalysis to alleviate the acidic and hypoxic tumour microenvironment and enhance pyroelectrodynamic therapy and mild PTT [129]. Similarly, AuPt‐loaded Fe‐N‐C nanocascade reactors with self‐supplied reaction substrates have been developed to improve catalytic performance in hypoxic tumours [128]. These studies suggest that the therapeutic benefit of CAT‐like nanozymes usually arises not from oxygen generation alone but from its integration with catalytic amplification, metabolic intervention, and other treatment modalities.

Beyond oxygen modulation, nanozymes can facilitate broader tumour microenvironment normalisation by regulating multiple metabolic and immunological pathways. These multifunctional agents typically exhibit complementary enzyme‐mimetic activities—including POD, GSHOx, and SOD—that collectively deplete antioxidants such as GSH, elevate ROS levels, and disrupt redox homeostasis. This cascade of events ultimately induces ICD while suppressing immunosuppressive cell populations [65, 82, 130, 131]. For example, advanced platforms such as PtFe@Fe_3_O_4_ and DMSN‐AuFe_3_O_4_‐CB839 integrate oxygen production with glutaminase inhibition, thereby augmenting oxidative stress while reversing immunosuppression [132, 133].

Tumour cells produce abundant lactate through the aberrant Warburg effect, leading to acidification of the tumour microenvironment. Beyond acting as a metabolic byproduct, lactate functions as an active immunometabolic signalling molecule that reinforces immune suppression through multiple layers of regulation. Mechanistically, lactate can be transported into macrophages via monocarboxylate transporters, where it stabilises HIF‐1α and induces the expression of VEGF, ARG1, and other M2‐associated genes, thereby promoting pro‐angiogenic and immunosuppressive TAM polarisation [134, 135]. In antigen‐presenting cells, lactate also restrains pro‐inflammatory signalling programs. In dendritic cells (DCs), lactate reduces the DNA‐binding activity of NF‐κB subunits, including p65, p50, and c‐Rel, at the IL‐12 promoter, thereby impairing IL‐12 production, DC maturation, and subsequent naïve T‐cell priming [136]. In macrophages, lactate signalling through GPR81 suppresses YAP‐dependent NF‐κB activation and pro‐inflammatory cytokine production, further skewing the TIME toward immune tolerance [137]. Beyond receptor‐ and transporter‐mediated signalling, lactate also exerts longer‐lasting immunoregulatory effects at the epigenetic level. At the epigenetic level, lactate serves as a substrate to induce histone lactylation, thereby promoting M2‐like gene expression during M1 macrophage polarisation and sustaining their long‐term suppressive phenotype [138]. Moreover, lactate accumulation suppresses CTL function within the TIME [139], while simultaneously reinforcing Tregs fitness and suppressive function [140]. In highly glycolytic tumours, lactate uptake through MCT1 promotes PD‐1 expression in Tregs [140], and lactate‐dependent MOESIN lactylation further enhances TGF‐β signalling, collectively consolidating the immunosuppressive state of the TIME [141].

Accordingly, lactate‐targeting nanozymes and metabolic enzyme‐targeting platforms have been developed to counter these immunosuppressive effects by consuming lactate, relieving acidosis, reversing glycolytic dysregulation, and reshaping the immune landscape [11, 12]. Specifically, by interfering with lactate efflux, these nanozymes cause intracellular lactate accumulation. This accumulated lactate then synergises with nanozyme‐delivered lactate oxidase to enhance ROS production and initiate a self‐circulating multienzyme cascade, promoting continuous generation of H_2_O_2_ and •OH while consuming intracellular GSH [142, 143]. The consequent ROS surge induces DNA damage and leakage, thereby activating the cGAS‐STING pathway. Additionally, the catalytic consumption of lactate coupled with O_2_ production collaboratively reshapes the immunosuppressive TIME [144]. Through these coordinated mechanisms, multimodal remodelling promotes TAM polarisation from M2 to M1 phenotype, reduces Treg activity, and enhances infiltration of CTLs and DCs—effectively converting immunologically “cold” tumours into “hot” ones [8, 12, 145, 146].

Notable examples include multifunctional nanozymes such as IMZF and gCM@MnAu, which exhibit POD, SOD, and GSHOx activities that disrupt redox balance, promote ICD, and facilitate DC activation with CD8^+^ T cell infiltration [82, 147]. Furthermore, certain nanozyme systems engage the STING pathway through Mn^2+^ ion release or ROS‐mediated DNA damage, thereby amplifying type I interferon signalling and potentiating innate immune responses [61, 79, 148].

The synergistic combination of hypoxia‐alleviating nanozymes with conventional therapies and immunotherapeutic agents enhances antitumour efficacy while promoting systemic immune responses. For example, metal ion‐releasing nanozymes (e.g., Mn^2+^) activate the cGAS‐STING pathway, amplifying innate immune signalling and enhancing DC maturation [149, 150]. When integrated with ICB such as anti‐PD‐1/PD‐L1 antibodies, these strategies can inhibit primary tumours and, in some preclinical bilateral or metastatic tumour models, suppress distant lesions or metastatic burden [8, 151, 152]. Accordingly, the current evidence is better described as system‐specific enhancement of systemic antitumour responses, rather than a universal improvement in abscopal effects across nanozyme platforms. Furthermore, the inherent theranostic capability of nanozymes—enabling real‐time imaging through modalities like MRI or photoacoustics—facilitates clinical translation by permitting precise monitoring of tumour microenvironment reoxygenation and treatment response [127, 153, 154, 155, 156]. In summary, the coordinated actions of oxygen supply, redox modulation, and metabolic reprogramming collectively enable broad tumour microenvironment normalisation and immune potentiation.

### 3.5 Immune Cell Modulation by Nanozymes

Nanozymes have emerged as powerful agents for modulating immune cell function within the TIME by remodelling immunosuppressive conditions and enhancing antitumour immunity. For example, Pt‐loaded hollow polydopamine (P@HP) and MnO_2_‐based nanozymes alleviate hypoxia through catalytic decomposition of H_2_O_2_ into oxygen, which reverses immunosuppression and promotes infiltration and activation of CTLs and M1‐type macrophages [145, 157]. Additionally, SnSe nanosheets mimic lactate dehydrogenase to metabolise lactate, leading to reduced acidity and restored T‐cell function [158]. Apart from targeting lactate, nanozymes can modulate other immunometabolic pathways. For instance, by degrading kynurenine or inhibiting indoleamine 2,3‐dioxygenase/tryptophan 2,3‐dioxygenase (IDO1/TDO) in the tryptophan‐kynurenine axis, they alleviate T‐cell suppression and enhance effector functions [159, 160]. Similarly, nanozymes that deplete ATP via purine metabolic interference—such as those catalysing ATP hydrolysis—reverse adenosine‐mediated immunosuppression and increase sensitivity to immune checkpoint inhibitors [161, 162].

Beyond metabolic reprogramming, nanozymes also activate antitumour immunity through inducing ICD and directly regulating immune cell populations. Copper‐based inducers and carbon quantum dots (CQDs), for instance, trigger ferroptosis, cuproptosis, or pyroptosis in cancer cells. This cascade recruits tumour‐infiltrating lymphocytes and reshapes the immune landscape, thereby converting immunologically “cold” tumours into “hot” ones and activating systemic antitumour immunity [163, 164, 165]. Simultaneously, such nanozymes can directly target immunosuppressive cells: dual‐targeting nanocarriers functionalised with specific peptides reduce Tregs and repolarise M2 macrophages toward the pro‐inflammatory M1 phenotype [145]. Moreover, nanozymes like Cu‐DBCO/CL downregulate immune checkpoint molecules (e.g., PD‐1, TIM‐3) on T cells and degrade extracellular matrix components, thereby enhancing T‐cell infiltration and cytotoxicity [166].

Nanozymes further potentiate antitumour immunity by activating key innate immune pathways and reprogramming macrophage polarisation. Mn‐based nanozymes, for example, activate the cGAS–STING pathway, augmenting CD8^+^ T‐cell infiltration and IFN‐γ secretion [13, 167]. Multifunctional systems such as Cu_2_O@CaCO_3_ not only induce tumour cell death via ROS and photothermal/photodynamic effects but also reprogram macrophages from the immunosuppressive M2 to the antitumour M1 phenotype. This dual activity bolsters systemic immunity and improves checkpoint blockade efficacy [168]. Other advanced nanozyme designs enhance ICD, relieve immunosuppression, and foster immune memory, collectively supporting sustained antitumour responses [169, 170].

Integrating nanozymes with established immunotherapies—such as ICB and adoptive cell transfer—synergistically enhances antitumour efficacy and overcomes therapeutic resistance. Nanozyme‐induced ICD and TIME remodelling markedly improve responses to anti‐PD‐1/PD‐L1 therapy by increasing immune cell infiltration and reversing adaptive immune resistance [76, 171]. In chimeric antigen receptor T‐cell therapy, nanozyme‐based delivery systems enhance gene editing efficiency and mitigate T‐cell exhaustion through targeted inhibition of immunosuppressive pathways [172]. Furthermore, the incorporation of multimodal imaging modules enables real‐time monitoring of immune activation and treatment response, facilitating the development of personalised therapeutic regimens [173].

In summary, nanozymes represent a versatile and innovative platform for immune modulation in cancer therapy. Through their enzyme‐mimetic activities, they precisely manipulate the TIME, induce ICD, reprogram immune cell phenotypes, and synergise with conventional immunotherapies. These integrated strategies collectively broaden and sustain antitumour immune responses, offering a promising avenue to overcome limitations of existing treatments.

## 4 Integrative Applications of Nanozymes in Combinatorial Cancer Immunotherapy

Nanozymes hold broad application potential in combinatorial cancer immunotherapy, where they act synergistically with various treatment modalities to enhance anti‐tumour immune responses. The combined therapeutic strategies employing nanozymes, along with their immune‐related synergistic mechanisms, are highly diverse. These include integration with ICB, CDT, phototherapy, conventional chemotherapy and radiotherapy, as well as other advanced platforms. Such multimodal approaches potentiate the efficacy of immunotherapy through multiple pathways, such as inducing ICD, remodelling the TIME, promoting antigen presentation, and enhancing immune cell infiltration. To facilitate cross‐study comparison, representative nanozyme‐based combinatorial immunotherapy studies are summarised in a standardised format including tumour model, dosing schedule, immune readouts, primary endpoint, and key safety findings (Table 3). The representative combinatorial strategies summarised in Table 3 are further discussed in the following sections.

**TABLE 3:** Representative studies of nanozyme‐based combinatorial immunotherapy.

Therapeutic strategy	Nanozyme system	Tumour model	Dosing schedule	Immune readouts	Primary endpoint	Key safety findings
Immune checkpoint blockade (ICB)	FeSA‐Ir@PF NSs [10]	Lung metastasis models	2 mg/mL + 10 mg/kg αPD‐L1+ 1064 nm laser	CD4^+^/CD8^+^T cell↑; IFN‐γ, IL‐6↑; DAMPs release	99.1% primary TGI; complete inhibition of lung metastasis nodules	No significant weight loss; no weight toxicity
Immune checkpoint blockade (ICB)	Cu_2_O‐OMV [9]	Renal cell carcinoma	3 mg/mL + 10 mg/kg αPD‐L1	CD80^+^, CD86^+^↑; CD8^+^T cell↑; IFN‐γ, TNF‐α↑	Significant TGI of primary and distant tumours	H&E: no organ abnormalities; Normal body weight
Chemodynamic therapy (CDT)	RuTe_2_‐GOx‐TMB [174]	4 T1 tumour‐bearing mice; lung metastasis model	i.t. injection on day 5 + 808 nm laser on day 6	CD4^+^/CD8^+^ T cells↑; IFN‐γ, IL‐3↑	Strong primary tumour inhibition; inhibited lung metastasis/recurrence	No significant weight loss
Chemodynamic therapy (CDT)	MnO_2_@TPP‐PEG [5]	Subcutaneous tumour model	100 μL i.v. + 660 nm laser	—	Strongest tumour inhibition	Stable body weight; no obvious organ damage
Phototherapy (PDT/PTT/SDT)	CaCO_3_@Pt‐TiO_2_ [81]	4 T1 tumour‐bearing mice	10 mg/kg i.v. + US	CD8^+^ T cells↑; TNF‐α, IL‐12↑	Strong tumour growth inhibition	No significant weight loss; no obvious major‐organ damage
Phototherapy (PDT/PTT/SDT)	HAuPt@Ce_6_‐PEG‐SH [175]	Breast tumour model	5 mg/kg + 808 nm/660 nm lasers	M1 TAMs↑/M2 TAMs↓; CD4^+^/CD8^+^ T cells↑; TNF‐α, IFN‐γ↑	Strong inhibition of primary and distant tumours; showed best tumour suppression and prevented recurrence	Stable body weight; no significant physical harm
Traditional chemotherapy and radiotherapy	Zn/Cu‐BSAN‐Dox NPs [176]	DOX‐resistant 4 T1 breast tumour model	i.v. injection; US at 6 h post‐injection	CD4^+^/CD8^+^ T cells↑; M1 macrophages↑	Strong tumour ablation; DOX resistance reversal; longest survival	No significant weight loss; no obvious major‐organ damage
Traditional chemotherapy and radiotherapy	GPGP [153]	Bilateral 4 T1 tumour model	i.v. injection +2 Gy X‐ray at 1 h	CD8^+^ T cells↑; M1 macrophages↑; M2 macrophages↓	93.8% primary TGI; prolonged survival; strong secondary tumour inhibition	No significant weight loss; no obvious organ damage
Other platforms	PBVac [177]	Melanoma postoperative recurrence model	10 mg/kg s.c. on 1,3,7 days+808 nm laser	DC maturation↑; CD4^+^/CD8^+^ T cells↑	Strong recurrent tumour inhibition	No significant weight loss; no obvious liver/kidney damage; normal blood biochemistry
Other platforms	DA‐CQD@Pd SAN hydrogels [178]	CT26 bilateral tumour model	Intratumoural injection at day 0; anti‐PD‐L1 i.v. on days 1/4/7	CD8−/Tregs ratio ↑; IFN‐γ, IL‐6↑	75% survival at day 30	No significant weight loss

Abbreviations: DAMPs, damage‐associated molecular patterns; DC, dendritic cell; DOX, doxorubicin; H&E, haematoxylin and eosin; i.t., intratumoural injection; i.v., intravenous injection; IFN‐γ, interferon‐γ; IL, interleukin; s.c., subcutaneous injection; TAM, tumour‐associated macrophage; TGI, tumour growth inhibition; TNF‐α, tumour necrosis factor‐α; Treg, regulatory T cell; US, ultrasound; αPD‐L1, anti‐programmed death‐ligand 1 antibody.

### 4.1 Nanozyme‐Enhanced Immune Checkpoint Blockade

Immune checkpoint blockade (ICB) has revolutionised cancer immunotherapy by blocking inhibitory receptors such as PD‐1 and CTLA‐4, thereby reactivating T‐cell‐mediated tumour killing [179]. This approach often leads to durable clinical responses in multiple cancer types, including melanoma, non‐small cell lung cancer, and hepatocellular carcinoma, owing to the establishment of immunological memory [180, 181]. Notably, in advanced melanoma, combination therapy with anti‐PD‐1 and anti‐CTLA‐4 antibodies has achieved a 5‐year overall survival rate of 52%, which was higher than that observed with either monotherapy [182]. Despite these successes, the efficacy of ICB is limited in immunologically “cold” tumours, which are characterised by poor T‐cell infiltration and an immunosuppressive microenvironment [183].

To address this limitation, nanozymes offer a promising strategy to convert these cold tumours into ICB‐responsive “hot” tumours by enhancing immunogenicity and remodelling the TIME. For instance, acoustic‐sensitive nanozymes (HMME/R837@Lip) combine SDT with PD‐L1 blockade and reduce tumour growth and metastatic burden in preclinical models [67]. At the mechanistic level, nanozymes enhance ICB by promoting ROS‐mediated ICD, thereby facilitating T cell activation and DC maturation [60, 184]. Copper‐based CGBH NNs exemplify this by disrupting glucose and redox homeostasis, which leads to disulfidptosis‐augmented pyroptosis and significantly enhances the response to αPD‐1 therapy in murine models [164]. Furthermore, the FeS@GOx hybrid nanozyme amplifies ROS through enzymatic cascades and suppresses antioxidant pathways, strengthening CDT and ICD (Figure 5A) [185]. Surface modifications—such as cancer cell membrane coatings—further improve tumour‐specific targeting and localised catalytic activity, consequently reducing off‐target effects [186].

**FIGURE 4:**
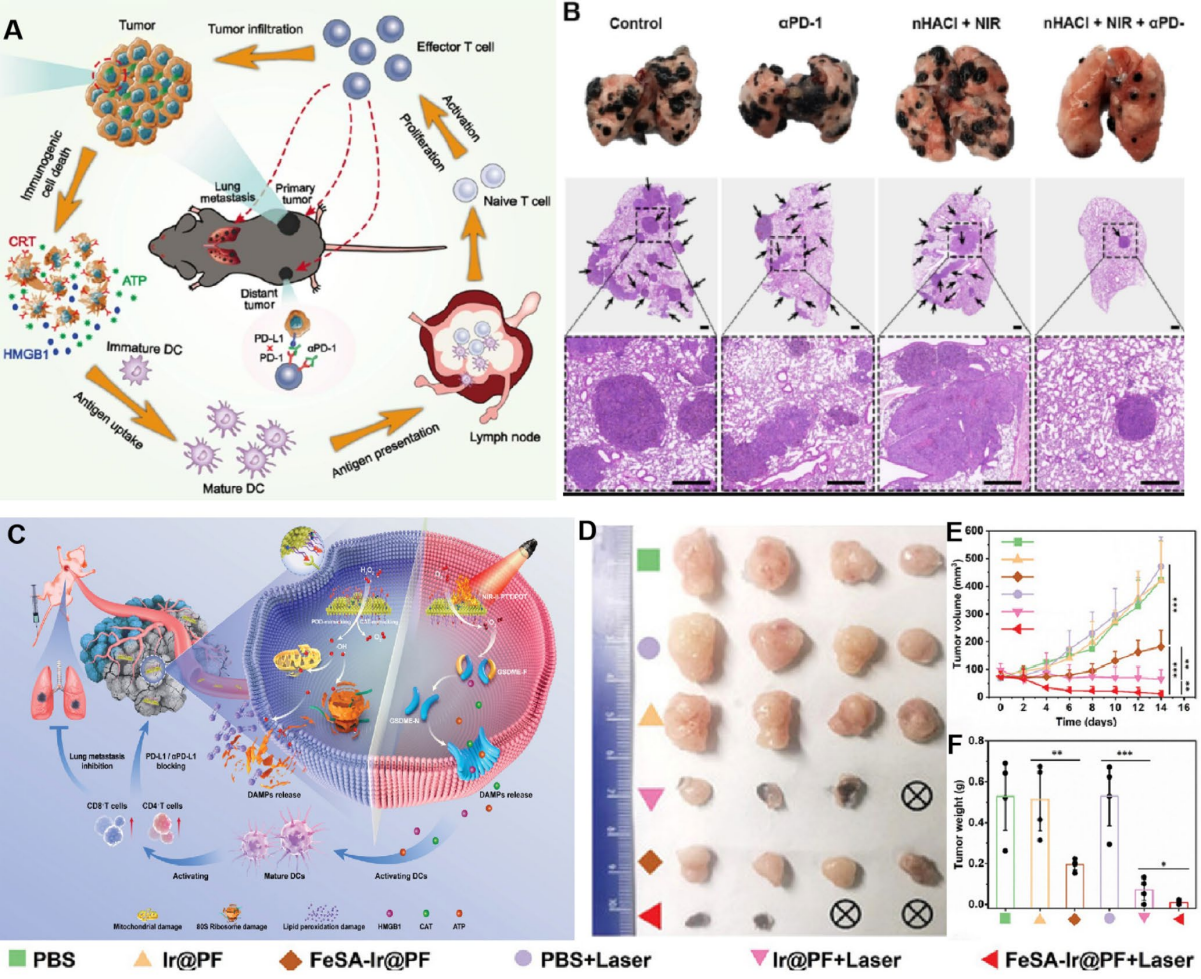
Nanozyme‐based combinatorial strategies to potentiate immune checkpoint blockade. (A) Schematic illustration of the fabrication of HA@Cu(OH)_2_–ICG (nHACI) and its cooperative anticancer mechanism combining photodynamic therapy (PDT) with PD‐1 blockade. (B) Representative lung photographs and H&E‐stained sections from differently treated mice. Mice treated with nHACI + NIR + αPD‐1 showed significantly fewer pulmonary nodules, indicating effective suppression of tumour metastasis. (A, B) Reproduced with permission [187]. Copyright 2022, Elsevier. (C) Schematic of FeSA‐Ir@PF NSs–induced ferroptosis in cancer cells; under light irradiation, synergistic PTT/PDT/CDT triggers pyroptosis. Guided by NIR‐II photoacoustic imaging, this system achieved 99.1% primary tumour eradication. (D) Photographs of tumours harvested after 14 days of treatment. (E) Individual tumour growth curves. (F) Average tumour weights after different treatments. (C–F) Reproduced with permission [10]. Copyright 2024, Wiley.

These combinatory approaches have shown compelling preclinical outcomes, including suppression of primary and metastatic tumours, induction of long‐term immune memory, and abscopal effects. For example, copper‐coordinated nanozymes (nHACI) enhance intracellular oxidative stress through their triple enzyme‐mimicking activities (POD, GSHOx, and CAT‐like), thereby potently inducing ICD. When used in combination with PD‐1 blockers, this strategy not only downregulates immunosuppressive M2 macrophages but also establishes long‐term immune memory, thereby effectively preventing tumour recurrence(Figure 4A,B) [187]. Furthermore, the remote effects of nanozyme‐based therapies have been validated in bilateral tumour models. By combining targeted nanozymes (ZPA) with ultrasound and anti‐PD‐L1 therapy, localised severe tissue damage can trigger the systemic release of large amounts of pro‐inflammatory cytokines, including TNF‐α, IL‐6, and IFN‐γ. This systemic immune cascade drives CD8^+^ CTLs to infiltrate large numbers of distant, unirradiated tumour beds, effectively eradicating distant lesions and completely suppressing invasive lung metastases [188]. Similarly, a biomimetic Cu_2_O‐OMV nanozyme induces dual apoptosis and pyroptosis, enhancing CTL infiltration and synergising with αPD‐L1 in renal cell carcinoma models [9]. In another study, the FeSA‐Ir@PF NSs system achieves sequential ferroptosis and pyroptosis under light control, leading to 99.1% primary tumour inhibition when combined with ICB (Figure 4C–F) [10]. Collectively, these innovative strategies highlight how nanozymes can overcome ICB resistance by transforming immunologically inert tumours into T‐cell‐inflamed environments.

### 4.2 Nanozyme‐Catalysed Chemodynamic Immunotherapy

Nanozymes have garnered significant attention for their ability to initiate self‐sustaining catalytic cascades that enhance CDT. A representative example is the RuTe_2_‐GOx‐TMB nanoreactor, which continuously consumes glucose and self‐supplies H_2_O_2_ through GOx activity, thereby sustaining RuTe_2_‐mediated Fenton‐like reactions and robust ROS generation while integrating starvation therapy, CDT, and PTT [174]. Beyond direct tumouricidal effects, the immunotherapeutic relevance of this self‐sustaining cascade lies in its ability to induce ICD and remodel the TIME. Specifically, excessive ROS production can trigger oxidative stress‐mediated tumour cell death, accompanied by the exposure of calreticulin and the release of DAMPs, including HMGB1 and ATP [189]. These signals promote dendritic‐cell maturation and antigen presentation, thereby facilitating the priming and activation of CD8^+^ cytotoxic T cells [30, 189]. In parallel, intensified oxidative stress and synergistic PTT‐mediated tumour ablation may help reduce local immunosuppressive signalling, while related cascade nanozyme systems have been reported to increase CD8^+^ and CD4^+^ T‐cell infiltration and reduce Treg prevalence in tumours [124, 189]. Therefore, the RuTe_2_‐GOx‐TMB system is not only a catalytic amplifier for CDT, but also a potential immune‐activating platform that links self‐sustaining oxidative damage to enhanced T‐cell‐mediated antitumour responses.

**FIGURE 5:**
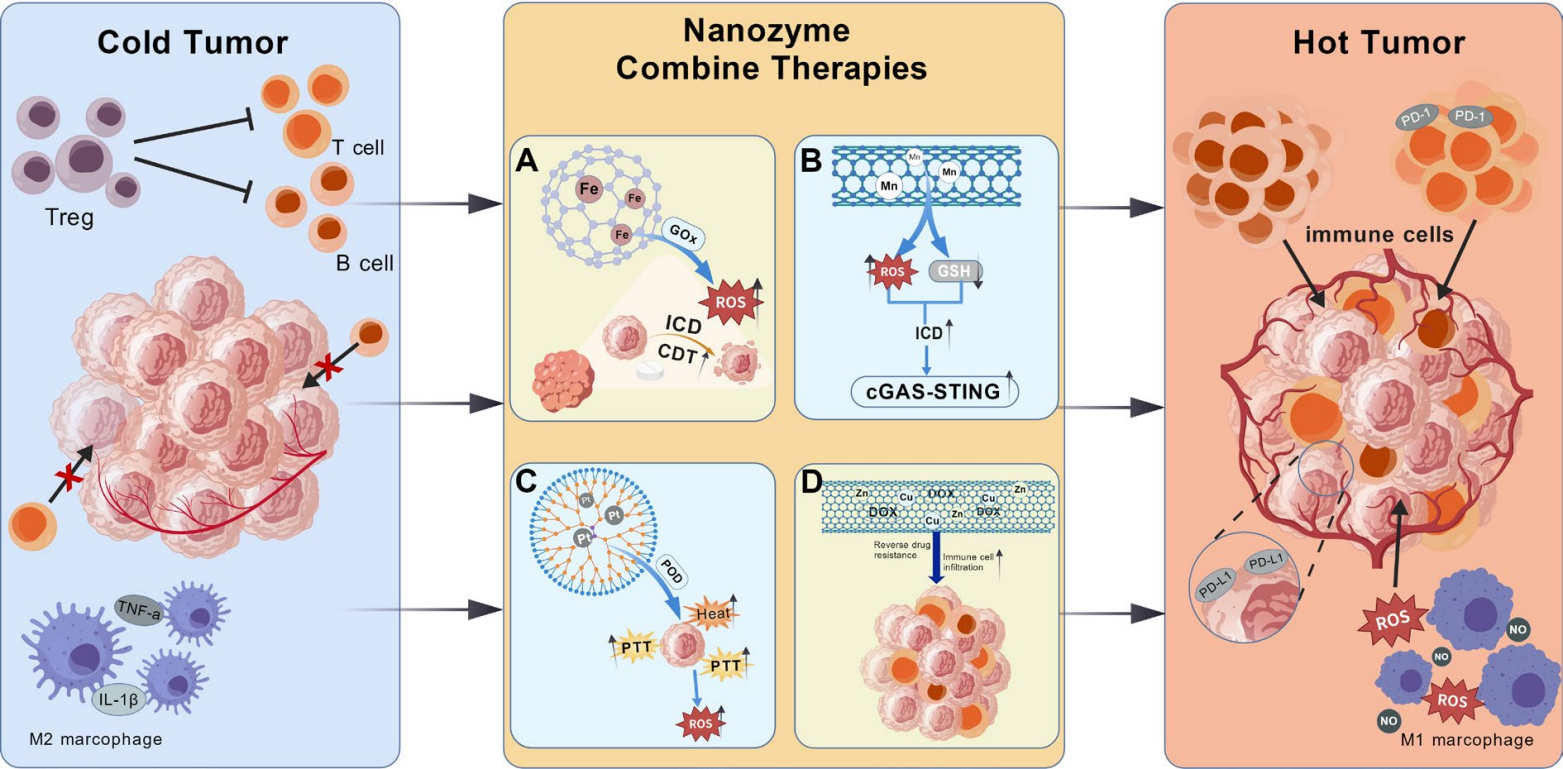
Nanozyme‐based combinatorial strategies for converting cold tumours into hot tumours. Illustrated are representative nanozyme systems incorporating (A) Fe, (B) Mn, (C) Pt, and (D) Cu/Zn in combined tumour therapy. Cold tumours are characterised by low immune infiltration, Treg‐mediated immunosuppression, and M2 macrophage predominance. Nanozymes facilitate reactive oxygen species (ROS) generation, glutathione (GSH) depletion, and immunogenic cell death (ICD), thereby activating the cGAS–STING pathway and augmenting chemodynamic (CDT), photothermal (PTT), and chemotherapeutic efficacy. These processes drive M1 macrophage polarisation, enhance T cell infiltration, and upregulate PD‐L1 expression, collectively remodelling the TIME into an immunologically active, “hot” tumour phenotype amenable to immunotherapy. Created with BioGDP.com.

Similarly, MnO_2_@TPP‐PEG catalyses H_2_O_2_ decomposition into O_2_ and ·OH, thereby mitigating hypoxia while simultaneously enhancing CDT and photodynamic efficacy (Figure 5B) [5]. Beyond this, other monometallic and multi‐enzyme nanozyme systems, together with MoS_2_/Co_3_S_4_@PEG heterostructures, also demonstrate efficient cascade catalysis and multimodal synergy with PTT, PDT, or SDT [154, 190, 191]. Complementing these approaches, GOx‐loaded and dual‐enzyme cascade nanozyme systems further enhance CDT by self‐supplying H_2_O_2_ and depleting nutrients [192, 193].

Building on these catalytic and immune‐activating effects, subsequent nanozyme platforms have further expanded CDT‐based immunotherapy through dendritic‐cell activation, macrophage repolarisation, and checkpoint blockade sensitisation. For instance, dual‐catalytic oxide nanosponges (DON) enhance ROS production under magnetic fields, promote DC maturation, and suppress metastasis when combined with anti‐PD‐1 [102]. Other strategies focus on modulating the TIME; systems such as IM@iPPAE@siMCT4 inhibit lactate export to reinforce CDT and promote M1 macrophage polarisation [146]. Furthermore, nanozymes including MnPt and Mn/Fe‐MIL‐101/CuS/DOX@FA induce ICD and reverse immunosuppression through coordinated ROS generation and GSH depletion [89, 194]. The immunostimulatory role of nanozymes is also evident as manganese‐based variants activate the cGAS‐STING pathway [195], while MDPH nanozymes combine ferroptosis induction with ICB to inhibit metastasis [118]. Expanding the scope, systems like CMZM, Zn/Cu‐BSAN‐DOX, Au/CuNDs‐R848, and PdPtCu nanozymes modulate oxygen metabolism, reverse drug resistance, induce in situ vaccination, and block immune escape, collectively underscoring the potent synergy between CDT and immunotherapy [176, 196, 197, 198]. CpG/Cu‐LDHs and gCM@MnAu nanozymes enhance ICD and improve PD‐1/PD‐L1 blockade efficacy, underscoring the clinical potential of CDT‐immunotherapy combinations [147, 199].

To overcome persistent therapeutic resistance and achieve multi‐pathway killing, novel nanozyme designs with integrated functions are being developed. A prominent example, FeCP@PDA‐GOx, integrates multiple enzyme activities to synergise CDT, starvation therapy, mild PTT, and immunotherapy while concurrently inducing ferroptosis [200]. In a targeted approach, HCuS/Au–Pt@DSF/ICG@9R–P201 depletes GSH and amplifies ROS to induce copper‐mediated cell death, demonstrating specific efficacy against hepatocellular carcinoma [201]. Multimodal systems further illustrate this trend; for example, Fe_3_O_4_@ZIF‐8/GOx@MnO_2_ and Co_3_O_4_@Ti_3_C_2_Tx couple CDT with starvation therapy, PTT, SDT, and immunotherapy through sophisticated multi‐enzyme or heterojunction designs [202, 203]. Finally, representing a novel theranostic direction, MnO_2_‐x/HPB mediates sonodynamic immunotherapy via cascaded ROS generation and cGAS‐STING activation [148].

### 4.3 Photo/Sono‐Responsive Nanozymes for Immune Activation

Phototherapy—encompassing PDT, PTT, and photoimmunotherapy—is widely utilised for its minimal invasiveness and tissue‐sparing properties [204]. Nevertheless, limitations such as inadequate immune activation and poor tissue penetration have motivated the development of nanozyme‐based combination strategies.

In PDT, nanozymes such as MnO_2_, MoS_2_/Co_3_S_4_, and ZnO alleviate hypoxia via H_2_O_2_ decomposition or photocatalytic oxygen generation, thereby significantly improving therapeutic outcomes [5, 154, 205, 206]. Beyond addressing hypoxia, multimodal platforms including GCS‐I‐PPy NZs, HAuPt@Ce6‐PEG‐SH, Pt–Mn–PEI, and CuCo_2_S_4_‐Pt‐PEG integrate PDT with PTT, CDT, and immune activation under single or dual‐wavelength irradiation [175, 207, 208, 209]. A distinct class of nanozymes, including nHACI, PdPtCu, and CMZM, focus on remodelling the TIME by inducing ICD, reversing immune escape, and modulating tumour metabolism [187, 196, 198]. Complementing these approaches, semiconductor polymer‐based SPNK nanozymes combine PDT with the release of kynureninase to degrade the immunosuppressive metabolite kynurenine, thereby boosting cytotoxic T‐cell infiltration [210]. Extending the principle of activation beyond light, the Au‐TiO_2_‐A‐TPP platform enhances ROS yield under ultrasound, bridging the application to SDT [211].

Transitioning to PTT, nanozymes such as RuTe_2_‐GOx‐TMB and FeCP@PDA‐GOx effectively integrate catalytic activity with thermal effects for robust immune activation [174, 200]. Other systems, including Ti_3_C_2_‐MXene‐Au, CuMnO_x_@ICG, Fe‐N‐C SAzyme, Pd@TiO_2_, and HAuHbO_2_, demonstrate synergistic PTT/CDT effects [26, 127, 193, 212, 213]. A paramount advantage of nanozymes in this context is their capacity for concurrent immune microenvironment remodelling and immunotherapy augmentation. A prime example is HAuPt@Ce6‐PEG‐SH, which simultaneously promotes macrophage M1 polarisation and ICD while enhancing the efficacy of combined PDT and PTT [175]. A wide array of other platforms—such as CuSR4M, IM@iPPAE@siMCT4, and Au/CuNDs‐R848—enhance antitumour immunity through mechanisms like tumour antigen release, lactate depletion, and myeloid‐derived suppressor cell (MDSC) suppression [107, 146, 197]. To directly combat the hypoxic and metabolic barriers in tumours, MoS_2_/Co_3_S_4_@PEG and CuCo(O)/GOx@PCN generate oxygen in situ to restore enzymatic activity and improve therapeutic efficacy [154, 214]. Furthermore, hollow Au‐Pt nanoshells leverage photothermal effects to boost their own CAT‐like activity, thereby reversing drug resistance (Figure 5C) [215], whereas Fe_3_O_4_@ZIF‐8/GOx@MnO_2_ combines oxygen generation with glucose consumption for enhanced CDT and starvation therapy [202]. Concluding the PTT strategies, theranostic systems such as UCNP‐based heterostructures enable imaging‐guided PTT/SDT with concurrent immune activation [216, 217], and systems like IR 820‐macrophage/GOx integrate mild PTT with pyroptosis induction to improve immunotherapeutic outcomes [218, 219].

In SDT, the integration of nanozymes with catalytic and immunomodulatory strategies is a key focus for amplifying treatment efficacy. Several systems, including Fe‐MoO_2_@PEG and B‐Au‐Ag_2_S‐HA, enhance ROS generation by exhibiting CAT‐like and glucose‐oxidase activities [220, 221]. Others, like Ni‐CoO@PEG, utilise oxygen vacancies to improve ROS production and multi‐enzyme functionality [222]. Beyond ROS‐centric mechanisms, alternative pathways are exploited: CaCO_3_@Pt‐TiO_2_ induces ICD through catalytic oxygen production and calcium ion release [81], and HABT‐C reverses immunosuppression via multi‐enzyme activity [223]. Immune remodelling is also achieved through distinct signalling pathways, exemplified by MnO_2_‐x/HPB via cGAS‐STING activation and Zn/Cu double single‐atom nanozymes via macrophage repolarisation [148, 176]. When combined with checkpoint blockade, the HMME/R837@Lip system improves SDT efficacy and inhibits metastasis [67]. Additionally, the PGI system amplifies outcomes through simultaneous oxygen generation and starvation therapy [152]. The theranostic potential of SDT nanozymes is highlighted by systems such as Ni‐CoO@PEG and MnO_x_ composites, which facilitate real‐time monitoring via magnetic resonance imaging [6, 222]. Moreover, Bi@Bi_2_O_3_–Pt‐PEG (BBOP) ternary heterojunctions employ Pt nanozymes to enhance sonocatalytic immunotherapy via improved oxygen supply [87].

In summary, nanozymes are revolutionising phototherapeutic and sonodynamic approaches by simultaneously mitigating tumour hypoxia, promoting robust and specific immune responses, and enabling real‐time theranostics.

### 4.4 Nanozyme‐Assisted Chemotherapy and Radiotherapy

Nanozymes are emerging as powerful adjuvants to traditional cancer therapies, including chemotherapy and radiotherapy. By modulating the tumour microenvironment, enhancing drug delivery, and inducing ICD, they effectively overcome key limitations of conventional treatments.

Representative examples demonstrate their utility in specific therapeutic contexts. For instance, deferasirox‐modified carbon dots (DFX‐CDs) chelate iron to induce ferroptosis and necroptosis, thereby improving anti‐PD‐L1 efficacy in triple‐negative breast cancer [224]. In radiotherapy, a gadolinium‐palladium platform (GPGP) with dual nanozyme activity enhances MRI‐guided radiotherapy while counteracting PD‐L1 upregulation [153]. Furthermore, the MFC system utilises iron‐doped nanozymes to produce oxygen under ultrasound, which alleviates hypoxia and enhances sonodynamic immunotherapy with αPD‐1 [225].

The synergistic potential of nanozymes with chemotherapeutics is particularly notable. A prominent example is doxorubicin (Dox) synergy: the 1T2H‐MoS_2_ nanozyme repolarises macrophages and promotes apoptosis, demonstrating a 3.4‐fold higher efficacy than Dox alone [226]. Beyond this, single‐atom nanozymes (Fe SAN‐PDA@DOX@HA) enable controlled drug release, GSH depletion, and ROS generation [130]. Zn/Cu‐BSAN‐DOX NPs function as “hexagonal warriors” to reverse drug resistance, enhance immune cell infiltration, and promote DC and M1 macrophage polarisation (Figure 5D) [176]. Biomimetic systems further expand this capability; for example, NMOF‐Fe/Cu‐Dox@M co‐delivers Dox and induces ferroptosis/calcification, thereby reshaping the TIME and improving ICB response [119]. Similarly, dNlgdPtx‐mHSA nanoparticles deliver paclitaxel and an IDO1 inhibitor to enhance immunogenicity [157]. Moreover, nitrogen‐doped carbon nanospheres with tumour microenvironment‐responsive dual enzymatic activity—synthesised via pyrolysis of covalent organic frameworks and loaded with Dox—significantly reduce chemotherapy cardiotoxicity while concurrently inducing ferroptosis and activating anti‐tumour immunity, offering a novel strategy for efficient and low‐toxic breast cancer treatment [227].

To address the challenge of cisplatin resistance, diverse nanozyme strategies have been developed. HA@MoCF_3_ NPs disrupt ATP7B and ERCC1 through reactive species generation, synergising with SDT [7]. Concurrently, CFe/βCP + Cis hydrogels facilitate the long‐term release of carbon nanozymes and cisplatin, effectively modulating the immune microenvironment [228]. UMON nanozymes release Mn^2+^ to produce ·OH and activate cGAS‐STING, while also protecting kidneys from ROS‐induced damage [195]. In a biomimetic approach, the CSD system enhances POD activity via cisplatin‐induced H_2_O_2_ upregulation, leading to mitochondrial damage and chemosensitisation [229]. Similarly, M1NV vesicles deliver dacarbazine (DTIC) to target M2 macrophages and induce tumour cell death [230].

Beyond direct tumour killing, nanozymes effectively target cancer stem cells (CSCs) and metastatic niches. Specifically, the MDPH nanozyme targets CD44‐positive CSCs, induces ferroptosis, and combines with αPD‐L1 to prevent metastasis [118]. Pt@PCN‐Cu disrupts mitochondrial function in pancreatic cancer and upregulates PD‐L1, thereby sensitising tumours to ICB [231]. Moreover, a core‐shell FM nanozyme co‐delivers gemcitabine and curcumin to induce ferroptosis, inhibit MDSCs, and activate cGAS‐STING, ultimately enhancing chemoimmunotherapy with dual‐mode MRI monitoring [13].

Collectively, these approaches illustrate how nanozymes can transform cold, resistant tumours into immune‐responsive environments, thereby significantly improving outcomes for traditional therapies.

### 4.5 Bioengineered Nanozyme Platforms for Advanced Immunotherapy

Nanozymes are increasingly integrated into innovative platforms—including vaccines, hydrogels, and biomimetic vesicles—to enable targeted and multifunctional approaches for enhancing cancer therapy.

**FIGURE 6:**
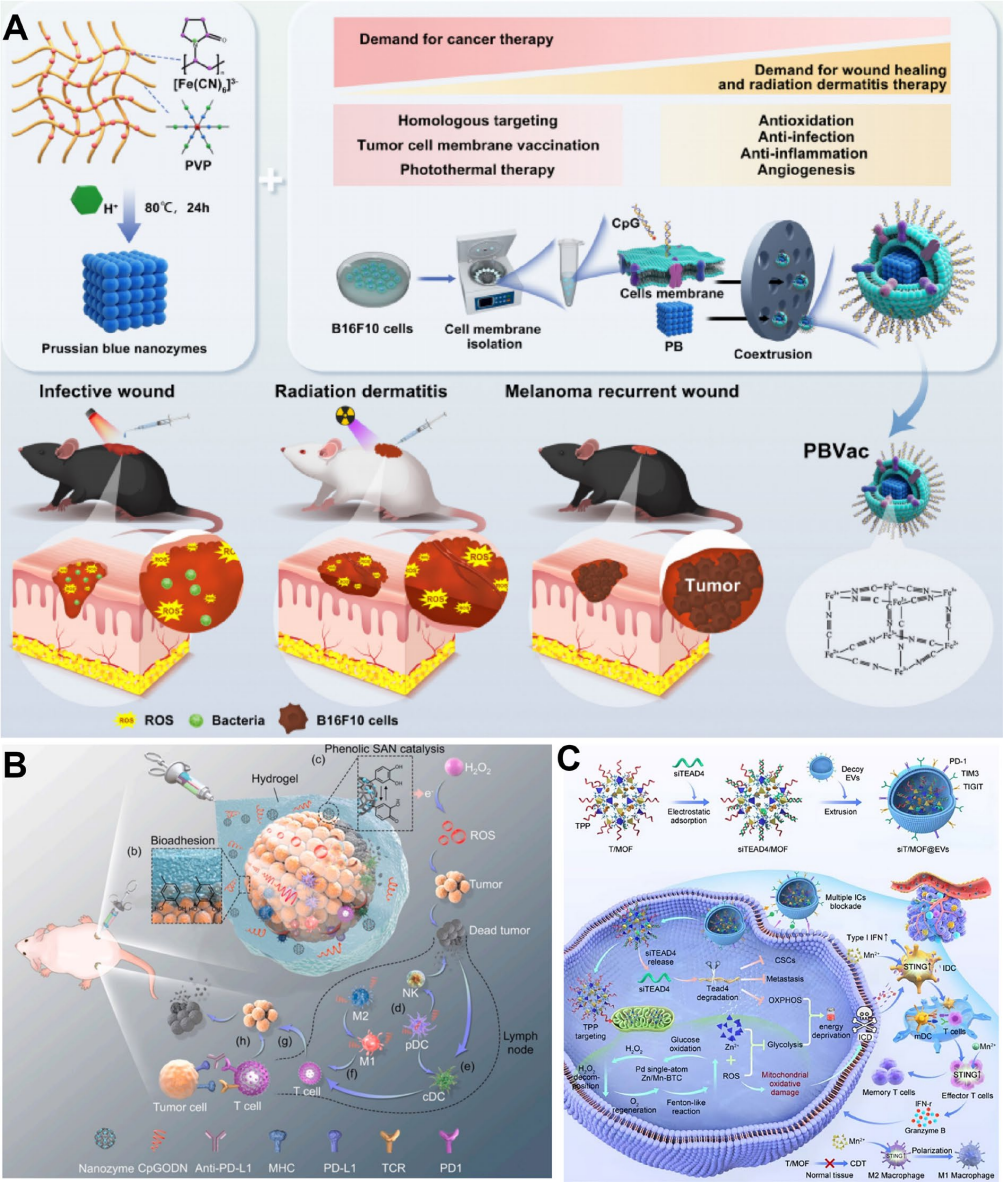
Bioengineered nanozyme platforms for advanced immunotherapy. (A) Schematic of PBVac for targeted tumour therapy. PBVac accumulates at tumour sites via homologous targeting and induces tumour eradication under laser irradiation. Tumour cell membranes are internalised by dendritic cells (DCs), leading to antigen presentation and T cell activation. The photothermal, antibacterial, and antioxidant properties of PB nanozymes also contribute to the therapeutic outcome. Reproduced with permission [177]. Copyright 2023, Elsevier. (B) DA‐CQD@Pd@CpGODN hydrogel enables catalytic immunotherapy against both primary and distant tumours. Reproduced with permission [178]. Copyright 2022, Elsevier. (C) siT/MOF@EVs overcome tumour heterogeneity in triple‐negative breast cancer and trigger an antitumour immune feedback loop with protective immune memory, mediated by a tumour cell membrane–cytoplasm–mitochondria cascade. Reproduced with permission [232]. Copyright 2025, Wiley.

In cancer vaccinology, nanozymes serve as potent enhancers of antigen delivery and immune activation. For instance, PBVac employs cell membrane camouflage for targeted delivery, concurrently integrating photothermal, antibacterial, and antioxidant functions (Figure 6A) [177]. Similarly, copper telluride nanozymes (CM CTNPs@OVA) induce ferroptosis and antigen release through PTT [233], while bimetallic AuCe nanozymes (ACO) enhance POD activity and inhibit PD‐L1 [234]. Furthermore, Dex‐HDL/ALA‐Fe_3_O4 not only generates antigens via PDT but also alleviates hypoxia [235]. Notably, HEzymes promote macrophage polarisation and reverse immunosuppression by virtue of their enhanced catalytic and photothermal performance [91].

Beyond vaccines, hydrogel systems facilitate the localised and sustained release of nanozymes, thereby improving therapeutic efficacy and reducing systemic toxicity. As illustrated in Figure 6B, the DA‐CQD@Pd SAN hydrogel not only generates ROS but also releases immune adjuvants, and its combination with anti‐PD‐L1 treatment effectively inhibits tumour metastasis [178]. In another example, the CFe/βCP + Cis hydrogel promotes ferroptosis and enhances CD8^+^ T‐cell infiltration [228]. Moreover, ZnO‐CuS/F127 hydrogels function by scavenging ROS, polarising macrophages, and promoting bone regeneration [236]. Additionally, MC@PHDA hydrogels integrate MoS_2_ nanozymes with photosensitisers to achieve PDT/PTT while providing liver protection [237]. Finally, Pd‐C single‐atom nanozyme hydrogels enable photothermal‐controlled drug release and self‐supply H_2_O_2_, which collectively enhance catalytic therapy and immune activation [238].

Similarly, biomimetic vesicle systems have been engineered to improve targeting specificity and immune modulation. For example, siT/MOF@EVs utilise a “bait” effect to block immune checkpoints and deliver siRNA and MOFs for inducing an oxidative burst (Figure 6C) [232]. In a parallel approach, MnNZ@OMV induces pyroptosis, leading to the activation of DCs and T cells [239], whereas Cu_2_O‐OMV enhances ICD by leveraging cuproptosis‐pyroptosis crosstalk [9]. RePd@OMVsPD‐L1 nb represents a more integrated strategy, combining POD/GSHOx activity with PD‐L1 inhibition for catalytic‐PTT [240]. Likewise, M1‐derived nanovesicles loaded with hMnOx reprogram TAMs to the M1 phenotype and induce ICD [230]. Furthermore, a platelet‐encapsulated copper single‐atom system (PPS) modulates metabolite levels to achieve self‐enhanced catalysis and metabolic therapy [241]. Collectively, these advanced platforms demonstrate the versatility of nanozymes in enabling precise, effective, and synergistic cancer therapies.

In summary, nanozymes represent a transformative tool in combinatorial cancer immunotherapy. Their ability to modulate the TIME, induce ICD, enhance conventional therapies, and synergise with immunotherapies collectively highlights their potential for converting immunologically “cold” tumours into “hot” ones, thereby paving the way for more effective and durable treatment strategies.

## 5 Conclusion and Future Perspectives

Nanozymes represent a class of groundbreaking catalytic nanomaterials capable of reprogramming the immunosuppressive TIME and converting immunologically “cold” tumours into “hot” ones, thereby revitalising the efficacy of cancer immunotherapy. Leveraging their multi‐enzyme mimetic activities—ranging from ROS generation and hypoxia alleviation to GSH depletion and metabolic interference—nanozymes can induce ICD, repolarise TAMs, promote DC maturation, and enhance CTL infiltration. Their ability to synergise with conventional therapies (e.g., chemotherapy, radiotherapy), phototherapies (PDT/PTT/SDT), and immune checkpoint inhibitors underscores their role as powerful immunomodulatory enhancers in combined anti‐tumour strategies.

Unlike traditional small‐molecule drugs, nanozymes possess numerous intrinsic advantages, including high catalytic specificity, tunable multi‐enzyme activity, and the potential for tumour microenvironment‐responsive activation. These features allow for increased therapeutic precision while minimising off‐target toxicity [242, 243]. For instance, systems like Fe/Cu‐HPC@GOx/PEG utilise bimetallic catalytic centers to induce dual ferroptosis and cuproptosis, effectively remodelling the immunosuppressive TIME and demonstrating promising anti‐tumour effects in preclinical bladder cancer models [108]. Similarly, defect‐rich MoS_2_ nanozymes (1T2H‐MoS_2_) exhibit enhanced POD‐like activity and have been successfully used to repolarise TAMs in human breast cancer samples, highlighting their clinical immunomodulatory potential [226]. Furthermore, bioactive materials such as SOD&Fe_3_O_4_@ZIF‐8 and engineered GOx‐Fe^0^@ZIF‐8 show additional benefits, including anti‐oxidant, anti‐inflammatory, and analgesic effects, along with higher cascade catalytic efficiency and lower systemic toxicity [244, 245].

However, despite numerous advancements in tumour microenvironment intervention for cancer therapy, several challenges to the clinical translation of nanozymes persist, including pharmacokinetics, long‐term biosafety, precise tumour targeting, and the functional heterogeneity of the tumour microenvironment [246, 247, 248, 249]. First, pharmacokinetics and in vivo metabolic pathways represent critical factors limiting the clinical application of nanozymes. Nanozymes are typically cleared through organs such as the liver, kidneys, or spleen, and different material compositions (e.g., metal‐based versus organic nanozymes) exhibit markedly different metabolic rates and biodistribution profiles, which directly influence their therapeutic efficacy and safety [250, 251, 252]. Long‐term biosafety is another crucial concern during clinical translation, particularly with respect to metal ion release, oxidative stress diffusion, and potential immunotoxicity. In complex physiological environments, metal‐based nanozymes may undergo partial degradation or ion leaching, leading to abnormal accumulation of metal ions such as Fe^2+^, Cu^+^, or Co^2+^ in non‐tumour tissues, thereby disturbing systemic metal homeostasis and inducing cytotoxicity [253]. In addition, although the robust ROS‐generating capability of nanozymes can enhance tumour ablation, insufficient control over ROS production may cause nonspecific oxidative damage to neighbouring hepatocytes, vascular endothelial cells, and immune cells. Persistent or excessive ROS exposure may also impair mitochondrial function, activate inflammatory signalling pathways, and, in some cases, promote fibrotic processes in normal tissues [254, 255]. Moreover, nanozyme‐mediated immune modulation must be carefully balanced, as prolonged oxidative stress or metal ion exposure may impair antigen‐presenting cell function, reduce lymphocyte viability, and disrupt cytokine homeostasis, thereby triggering immunotoxic responses [256].

Importantly, the in vivo catalytic behaviour of nanozymes may differ substantially from that observed in simplified in vitro systems. In biological fluids, protein corona formation can shield catalytic active sites, interfere with substrate access, alter biodistribution, and attenuate effective catalytic performance [45]. In addition, the highly reductive intracellular milieu of tumours, particularly elevated GSH, may consume reactive intermediates, quench reactive oxygen species, or reduce catalytically active metal centers, thereby compromising catalytic performance in vivo. Consistently, biomolecular corona formation has also been shown to switch off the catalytic activity of platinum nanozymes in serum before their intracellular reactivation under specific lysosomal conditions, highlighting the strong context dependence of nanozyme function in vivo [257]. In addition, intracellular trafficking and endolysosomal processing may further influence nanomaterial escape, substrate accessibility, and catalytic persistence, thereby affecting the actual therapeutic output after cellular uptake [258]. Therefore, although nanozymes have shown encouraging preclinical efficacy, clinical validation of nanozyme‐based therapy remains limited, and the gap between proof‐of‐concept nanocatalytic therapy and reproducible therapeutic benefit in complex human tumours should be more carefully acknowledged.

Beyond these systemic pharmacological and biosafety concerns, the clinical performance of nanozymes also critically depends on whether they can accumulate efficiently and function selectively within tumour tissues. The accurate targeting and accumulation of nanocatalysts within tumours is therefore a critical issue, as off‐target distribution may not only reduce therapeutic efficiency but also aggravate systemic toxicity [259]. Moreover, the complexity and heterogeneity of the tumour microenvironment—including variable pH levels, severe hypoxia, and elevated GSH levels—can significantly diminish the catalytic activity and efficacy of nanozymes [260]. For instance, while CuCo_2_S_4_‐Pt‐PEG nanocomposites exhibit excellent photothermal and enzymatic performance under ideal conditions, their efficacy may be compromised in deeply hypoxic or heterogeneous tumours [209]. At a further translational level, these biological challenges are compounded by practical issues related to formulation engineering and manufacturing. In particular, the scalability, reproducibility, and long‐term stability of nanozyme formulations must be rigorously evaluated to meet Good Manufacturing Practice standards for clinical use. Recent multifunctional platforms (e.g., Cu@Fe_2_C@mSiO_2_‐R848‐ICG‐AS1411 and PtMnIr alloy nanozymes) exemplify innovative catalytic‐immune cooperation but also highlight issues associated with synthetic complexity and undefined in vivo metabolic pathways [260, 261]. The development of tumour vaccines may offer a promising future direction for enhancing the precise targeting of nanozymes [262]. Ultimately, future strategies must balance catalytic efficacy, biosafety, and clinical feasibility to achieve successful translation.

To overcome these obstacles, innovative strategies are being developed to enhance the specificity, safety, and functionality of nanozymes. Current research focuses on biomimetic surface modification, utilising coatings such as cancer cell membranes or macrophage‐derived vesicles to enhance tumour homing and immune compatibility [230]. Nanozymes like ultra‐small (UMON) exhibit “switchable” ROS generation and scavenging properties, potentially reducing side effects in normal tissues while enhancing tumour‐specific CDT [195]. Furthermore, integrating real‐time imaging modalities (e.g., MRI, photoacoustics) allows for precise monitoring of nanozyme distribution and catalytic activity, facilitating personalised treatment guidance and improving clinical applicability [197]. A nanozyme successfully targeting aberrant lactate metabolism in tumours enhanced lactate oxidase activity and played a role in reconstructing the immune‐suppressive microenvironment's homeostasis, highlighting future opportunities in remodelling immune‐metabolic homeostasis [263].

Building on these advances, an increasing number of recent studies have increasingly explored nanozyme‐triggered endogenous enzyme cascade reactions as a next‐generation therapeutic strategy. In this emerging paradigm, nanozymes function not only as independent catalytic units but also as catalytic initiators that activate or amplify endogenous enzyme pathways within the tumour microenvironment. For example, hydrogen peroxide or oxygen generated by nanozyme catalysis may further stimulate endogenous peroxidases or oxidases, thereby establishing a self‐amplifying catalytic cascade network that enhances ROS production, metabolic disruption, and immune activation [174, 261, 264]. Such cascade amplification may improve therapeutic efficacy while lowering the required nanozyme dose, which could, in turn, reduce systemic toxicity and enhance biosafety and translational feasibility. Moreover, the development of stimuli‐responsive nanozyme systems represents another highly promising direction. By integrating tumour‐specific microenvironmental cues—such as acidic pH, hypoxia, redox imbalance, or the overexpression of specific enzymes—nanozymes can be selectively activated within tumour tissues while remaining relatively inert in normal tissues [26, 265, 266]. This tumour microenvironment‐triggered and spatiotemporally controllable catalytic strategy is expected to markedly improve the selectivity, safety, and precision of nanozyme‐based therapy, thereby laying a stronger foundation for future clinical translation.

Looking ahead, the successful clinical implementation of nanozyme‐based therapies requires a balanced optimisation across catalytic efficacy, biosafety, and translational feasibility. Rational design strategies should emphasise biocompatibility, stimulus‐responsiveness, and tumour‐specific activation. Standardised characterisation protocols, rigorous preclinical validation, and well‐structured early‐phase clinical trials are paramount. Finally, synergistic collaboration across materials science, immunology, and oncology will be the key driver in pushing nanozyme‐combined immunotherapy from the laboratory bench to the clinical bedside.

In summary, nanozymes offer a versatile and transformative platform for the next generation of cancer immunotherapies. By harnessing their catalytic power to reshape the TIME and enhance anti‐tumour immunity, nanozymes may help overcome key limitations of existing therapies, bringing us closer to durable and systemic cancer control.

## Author Contributions

**Yue Wang:** conceptualisation, writing – original draft, writing – review and editing. **Miao Xu:** writing – original draft. **Yongshun Wang:** writing – original draft. **Xuan Lin:** resources. **Qiang Zhang:** writing – original draft. **Xiaoning Lin:** resources. **Xin Wu:** resources. **Cheng Zhang:** supervision, conceptualisation. **Wenhua Huang:** supervision, conceptualisation. **Jianlin Shen:** writing – review and editing, supervision, conceptualisation.

## Funding

This work was supported by the Natural Science Foundation of Fujian Province (No. 2025J08239), the Young and Middle‐aged Teachers Educational Scientific Research Program of Fujian Province (No. JAT241104), the Startup Fund for Advanced Talents of Putian University (No. 2024148), the Key Healthcare Projects of Putian (No. 2024SJYL043), the Medical Research Foundation of Putian University (No. 2024104), and the Science and Technology Bureau of Putian City (No. 2025SZ3001PTXY08).

## Ethics Statement

The authors have nothing to report.

## Conflicts of Interest

The authors declare no conflicts of interest.

## Data Availability

Data sharing not applicable to this article as no datasets were generated or analysed during the current study.
